# Luteolin for Alzheimer's disease and related cognitive impairment: a systematic review and meta-analysis of efficacy and molecular mechanisms

**DOI:** 10.3389/fmed.2026.1976119

**Published:** 2026-10-14

**Authors:** Jixuan Xin, Xilai Teng, Hongyan Su, Xiancheng Wang, Mingquan Li

**Affiliations:** 1 College of Integrated Traditional Chinese and Western Medicine, Changchun University of Chinese Medicine, Changchun, Jilin Province, China; 2 Department of Cardiology, The Third Affiliated Clinical Hospital of Changchun University of Chinese Medicine, Changchun, Jilin Province, China; 3 Department of Neurology, The Third Affiliated Clinical Hospital of Changchun University of Chinese Medicine, Changchun, Jilin Province, China

**Keywords:** Alzheimer's disease, luteolin, meta-analysis, neuroinflammation, oxidative stress, systematic review

## Abstract

**Background:**

The multifactorial pathogenesis of Alzheimer's disease (AD) renders conventional single-target therapies largely ineffective. Luteolin, a natural flavonoid, has demonstrated promising pleiotropic neuroprotective potential. This study aims to systematically evaluate the preclinical efficacy and multi-target mechanisms of luteolin in rodent models of AD and associated cognitive impairment.

**Methods:**

Eight major electronic databases were systematically searched from January 1, 2000 to June 1, 2026. *In vivo* preclinical studies evaluating luteolin monotherapy in AD-specific and general cognitive impairment rodent models were included. Methodological quality was assessed using the SYRCLE risk of bias tool. Standardized mean differences and 95% confidence intervals were calculated using a random-effects model.

**Results:**

Fifteen eligible studies were included. Meta-analysis revealed that luteolin significantly ameliorated cognitive and memory impairments in both AD-specific and non-AD cognitive impairment models. Mechanistically, luteolin significantly restored redox homeostasis and suppressed neuroinflammatory cascades at both transcriptional and translational levels. Subgroup analyses indicated that oral administration and prolonged interventions were associated with significant efficacy; however, these observations are heavily influenced by study-level confounding and cannot conclusively establish pharmacokinetic superiority. Furthermore, the certainty of evidence is limited by predominantly unclear risks of bias, substantial statistical heterogeneity, and potential publication bias.

**Conclusion:**

Current preclinical evidence suggests that luteolin is a promising multi-target neuroprotective candidate for AD and related cognitive impairments, highlighted by its anti-inflammatory and antioxidant properties. However, given the substantial methodological limitations and extremely large pooled effect sizes, these findings must be interpreted with caution. Future research must prioritize rigorous preclinical designs, advanced brain-targeted delivery systems, and GLP-compliant toxicological profiling before clinical translation.

## 1 Introduction

Alzheimer's disease (AD) is the most common cause of dementia in the elderly, and its clinical features are progressive cognitive decline and memory loss (1). As the global population ages rapidly, AD imposes a devastating socioeconomic burden on families and healthcare systems around the world (2). Currently approved drug treatments, such as cholinesterase inhibitors and NMDA receptor antagonists, can only temporarily relieve symptoms and cannot fundamentally prevent or reverse the underlying pathological progression. Although recent advancements in A*β*-directed monoclonal antibodies have demonstrated certain breakthroughs in slowing cognitive decline, their widespread clinical application remains substantially hampered by exorbitant costs and the risk of adverse events (3, 4). Because the pathogenesis of AD is highly complex—involving deeply interwoven networks of A*β* cascades, tau hyperphosphorylation, neuroinflammation, oxidative stress, and mitochondrial dysfunction—single-target interventions have consistently fallen short of yielding ideal clinical efficacy (5–7).

In this context, naturally occurring flavonoids have garnered considerable attention for their pleiotropic neuroprotective effects and relatively low toxicity profiles (8–10). Luteolin (3′,4′,5,7-Tetrahydroxyflavone) is a prominent natural flavonoid ubiquitously distributed in various dietary vegetables, fruits, and traditional medicinal plants. Extensive *in vitro* and *in vivo* studies have established that luteolin exhibits exceptional antioxidant, anti-inflammatory, anti-apoptotic, and neurotrophic activities (11). More importantly, as a lipophilic small molecule, luteolin has been demonstrated to effectively cross the blood-brain barrier (BBB) and distribute into the central nervous system, establishing a fundamental pharmacokinetic prerequisite for central neuroprotection (12, 13). This establishes a robust pharmacokinetic foundation for its development as a candidate drug for neurodegenerative diseases.

In recent years, multiple preclinical studies have explored the therapeutic potential of luteolin in various animal models of AD. Cumulative evidence indicates that luteolin can markedly improve learning and memory impairment in A*β*-infused models, transgenic mice and chronic cerebral hypoperfusion models. It also alleviates A*β* amyloid pathology, tauopathy and synaptic dysfunction via several signalling pathways, such as modulation of the NF-*κ*B inflammator*y* axis, activation of the PI3K/Akt pro-survival pathway, and PPAR*γ*-dependent mitochondrial protection. Despite these promising findings from individual experiments, notable methodological heterogeneity persists among published work with respect to animal strains, disease-induction protocols, drug dosages, delivery routes and outcome assessments. No rigorous quantitative synthesis has yet been published to assess the overall efficacy of luteolin for AD. In addition, its intricate multi-target mechanism and relevant translational barriers remain to be systematically summarised.

Given the pivotal bridging role of preclinical data in guiding translation toward human clinical trials, conducting a rigorous evidence-based evaluation is of paramount urgency. Therefore, the present study aims to conduct a comprehensive preclinical systematic review and meta-analysis to quantitatively evaluate the efficacy of luteolin interventions in AD animal models, while systematically delineating its underlying multi-target neuroprotective mechanistic network. This study seeks to provide high-quality evidence-based support for the druggability of luteolin as an anti-AD candidate compound and to offer critical insights for future formulation engineering optimization and the design of clinical trials.

## 2 Methods

This preclinical systematic review and meta-analysis was conducted strictly in accordance with the Preferred Reporting Items for Systematic Reviews and Meta-Analyses (PRISMA) guidelines. The prospective study protocol was registered in the International Prospective Register of Systematic Reviews (PROSPERO) under the registration number CRD420261423871.

### 2.1 Search strategy

Two investigators systematically searched eight major English and Chinese electronic bibliographic databases, including PubMed, Web of Science, Embase, The Cochrane Library, China National Knowledge Infrastructure (CNKI), Wanfang Database, VIP Database, and the Chinese Biomedical Literature (CBM) database. The search timeframe spanned from January 1, 2000, to June 1, 2026. The search strategy employed a combination of Medical Subject Headings (MeSH) and free-text terms, with core search components comprising “Alzheimer's disease”, “dementia”, and “luteolin”. No language restrictions were imposed during the literature search. To minimize the risk of omitting relevant literature, the reference lists of the included studies and relevant review articles were manually screened, and the search was updated prior to the final data analysis to capture any recently published studies (Supplementary Materials 1).

### 2.2 Eligibility criteria

The inclusion criteria were strictly formulated based on the PICOS (Participants, Intervention, Comparison, Outcomes, Study design) framework. Eligible participants (P) included *in vivo* rodent models (restricted to rats and mice) of AD and related cognitive impairments. To ensure comprehensive translational relevance, this overarching target population is explicitly intended to encompass both AD-specific pathological models and non-AD models of isolated cognitive impairment. To ensure high methodological clarity, the eligible rodent models were conceptually classified into two categories: (1) AD-specific models (e.g., transgenic constructs and A*β*-infusion); and (2) cognitive impairment models (e.g., scopolamine, LPS, D-galactose, traumatic brain injury, and chronic cerebral hypoperfusion). Because this study focuses on shared downstream pathological cascades—specifically oxidative stress and neuroinflammation—data from these varied etiologies were synthesized collectively to evaluate the broad-spectrum neuroprotective potential of luteolin. The intervention (I) required luteolin to be administered as the sole therapeutic agent in the experimental group, with no restrictions on dosage, route of administration, or duration of intervention. For comparison (C), studies had to include AD model animals receiving either an equal volume of a non-functional vehicle (e.g., normal saline, distilled water) or no treatment. Furthermore, the included studies (S), which were restricted to *in vivo* animal experiments, had to report at least one of the following predefined outcomes (O): cognitive and behavioral performance, core AD neuropathological hallmarks, or markers of neuronal survival, synaptic plasticity, oxidative stress, or neuroinflammatory cytokines.

Studies meeting any of the following criteria were excluded: (1) non-animal experiments, human clinical trials, case reports, reviews, conference abstracts, or editorials; (2) studies utilizing non-rodent animal models or evaluating non-AD neurological disorders; (3) interventions involving luteolin combined with other pharmacologically active components, precluding the isolation of luteolin's independent effects; (4) studies lacking a clear modeling methodology or a valid disease control group; (5) duplicate publications, or studies with incomplete data that remained irretrievable after attempting to contact the corresponding authors via email.

### 2.3 Data extraction

Two researchers independently extracted data using a standardized data extraction spreadsheet, followed by rigorous cross-verification. The extracted data primarily included the first author, publication year, baseline characteristics of the animals (species, sex, sample size), modeling mechanisms, luteolin administration regimens (dose, route, duration), and all predefined primary and secondary outcome measures. For studies presenting data exclusively in graphical formats, plot digitizer software (GetData Graph Digitizer, version 2.26) was utilized in a double-blind manner to extract coordinates.

Strategy for Unit-of-Analysis Error Correction: To mitigate unit-of-analysis errors arising from multiple intervention groups (e.g., varying luteolin doses) sharing a single model control group, we employed a conditional, two-tier strategy in accordance with the Cochrane Handbook (14). Primarily, the total sample size (*N*) of the shared control group was divided evenly among the active comparisons (while maintaining the original means and standard deviations) to prevent the inappropriate double-counting of control animals. However, if this division resulted in a fractional or critically small control sample size—which would inherently cause severe instability in variance estimation and compromise statistical power—we alternatively applied a mathematical merging approach. In such cases, the multiple luteolin dose groups were mathematically combined into a single composite intervention arm. The combined sample size (*N*_combined_), mean (*M*_combined_), and standard deviation (*SD*_combined_) for two groups were calculated using the following explicit formulas:

$$N_{\mathrm{combined}}=N_{1}+N_{2}$$

$$M_{\mathrm{combined}}=\frac{N_{1}M_{1}+N_{2}M_{2}}{N_{1}+N_{2}}$$

$$SD_{\mathrm{combined}}=\sqrt{\frac{(N_{1}-1)SD_{1}^{2}+(N_{2}-1)SD_{2}^{2}+\frac{N_{1}N_{2}}{N_{1}+N_{2}}{\left(M_{1}-M_{2}\right)}^{2}}{N_{1}+N_{2}-1}}$$

For studies with three or more dose groups meeting this criterion, the merging procedure was applied sequentially. Furthermore, to prevent statistical dependency, if multiple outcomes, distinct behavioral trials, or repeated time-point measurements were derived from the identical cohort of animals, we systematically extracted data only from the longest intervention time point or the predefined primary metric. This protocol ensures that multiple datasets from the exact same animals were never treated as independent observations within a single meta-analysis.

### 2.4 Quality assessment and risk of bias

Two researchers independently evaluated the methodological quality and risk of bias of the included literature utilizing the SYRCLE's risk of bias tool for animal studies. This assessment framework comprises 10 items spanning 6 domains of bias: selection bias, performance bias, detection bias, attrition bias, reporting bias, and other sources of bias. Each item was rated as having a “low risk”, “high risk” or “unclear risk”. “Other bias” was explicitly defined as any potential source of bias not covered by the preceding five domains, specifically including industry funding/sponsorship, baseline imbalances, or the use of inappropriate statistical methods. Inter-rater reliability between the two evaluators was quantitatively assessed using Cohen's kappa statistic. Any discrepancies were initially resolved through mutual discussion; if a consensus remained elusive, a third senior researcher was consulted for final arbitration.

### 2.5 Statistical analysis and visualization

All statistical analyses and data visualizations in this study (including meta-analysis forest plots, sensitivity analysis plots, and funnel plots) were uniformly programmed and generated using the R programming language. For continuous variable data, the standardized mean difference (SMD) and its 95% confidence interval (CI) were calculated as the pooled effect size. Given the inherent biological variability in animal experiments and the expected substantial heterogeneity across studies, a random-effects model was universally employed for data pooling. Heterogeneity was assessed using the Cochran's *Q* test and the *I*^2^ statistic; significant statistical heterogeneity was defined as *P* < 0.05 and *I*^2^ ≥ 50%. To explore potential sources of heterogeneity, pre-specified subgroup analyses were conducted based on administration dosage and intervention duration. Sensitivity analyses were performed using the leave-one-out approach to verify the robustness of the results. Potential publication bias was qualitatively evaluated via the visual symmetry of funnel plots, and quantitatively detected by executing Egger's regression test using R software (where *P* < 0.05 indicated significant publication bias).

## 3 Results

### 3.1 Literature search and study selection

Through a comprehensive and systematic search of the selected databases, a total of 2,223 relevant records were initially identified. After excluding 851 duplicate records, 1,372 articles remained. Further screening of titles and abstracts resulted in the exclusion of 1,352 records at this stage (of which 775 were irrelevant to the study topic, 522 were reviews or meta-analyses, and 55 did not use rat or mouse models). Subsequently, 20 reports were successfully identified and subjected to a rigorous assessment against the full-text inclusion criteria. Based on predefined exclusion criteria, an additional 5 reports were excluded (4 due to incomparable interventions and 1 due to the lack of predefined outcome measures). Ultimately, 15 eligible studies were formally included in this systematic review and meta-analysis (Figure 1).

**Figure 1:**
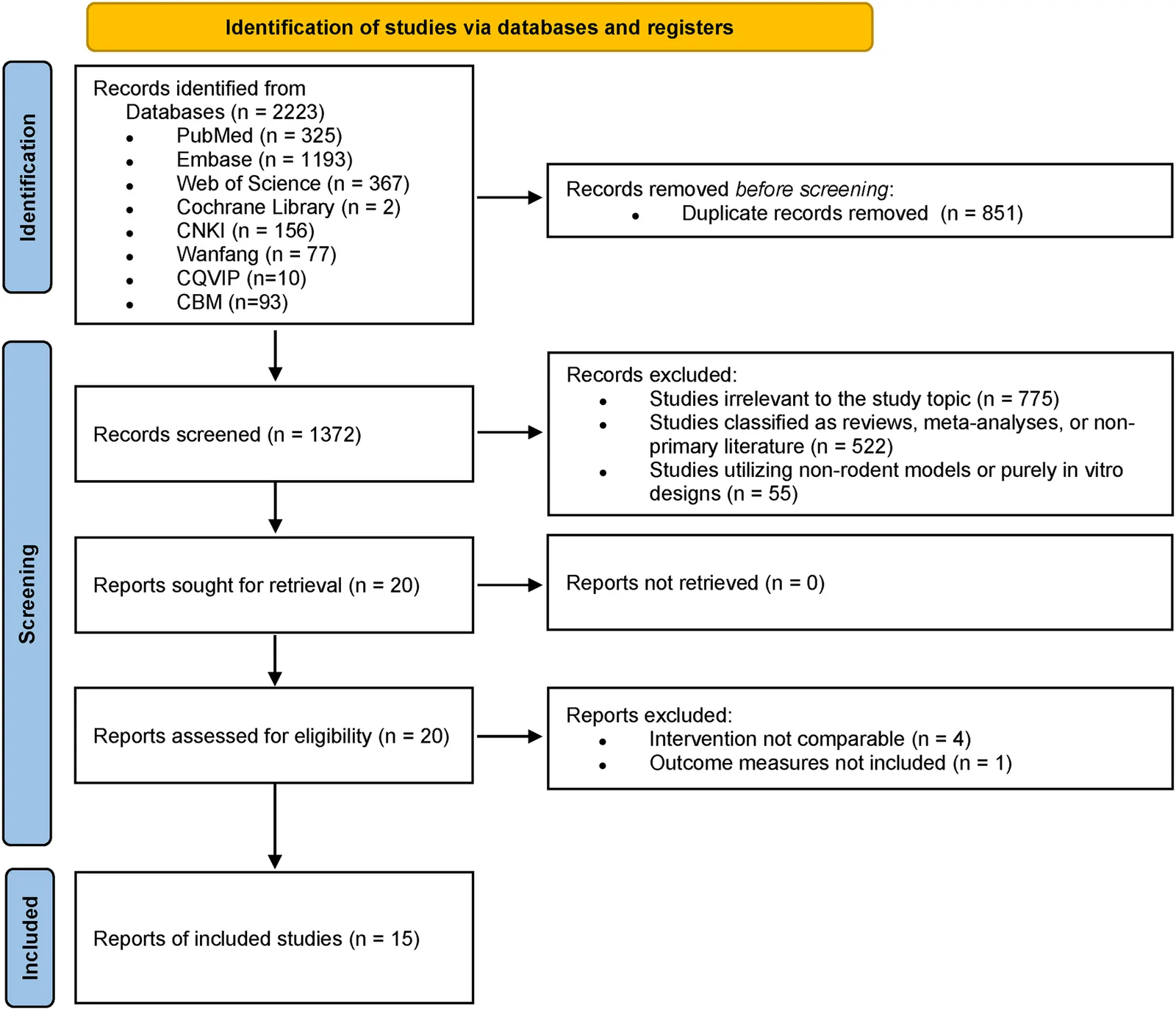
PRISMA 2020 flowchart for literature screening.

### 3.2 Study characteristics

This systematic review included a total of 15 preclinical studies in rodents; their main characteristics are summarized in Table 1 (15–29). Animal models included transgenic AD models, A*β* perfusion models, 2-VO chronic cerebral hypoperfusion models, traumatic brain injury models, D-galactose-induced aging models, and neuroinflammatory models of cognitive impairment mediated by scopolamine and LPS. The 15 included studies utilized both rats and mice. While individual baseline characteristics are detailed in Table 1, overall, the majority employed exclusively male cohorts (*n* = 10), followed by mixed-sex cohorts (*n* = 3) and female cohorts (*n* = 1). Sample sizes ranged from 8 to 20 animals per experimental group and were reported in all studies. However, critical baseline information was occasionally missing from the original literature: explicitly, 1 study failed to report animal sex or specific strain, and 4 studies failed to document the exact animal age or body weight. There was significant heterogeneity in the luteolin intervention protocols. Routes of administration included oral gavage, intraperitoneal injection, and dietary supplementation. The luteolin dosage range was 25–200 mg/kg in rats and 20–120 mg/kg in mice, with treatment durations ranging from 14 days to 3 months. Evaluation parameters primarily included behavioral and cognitive assessments, histopathological examinations, oxidative stress biomarkers, neuroinflammatory mediators, and molecules associated with signaling pathways.

**Table 1:** Study characteristics of included studies.

Study ID	Model	Sample	Luteolin intervention	Treatment duration	Anesthesia	Outcome measures
Ashaari et al. (15)	[Cognitive impairment] Wistar rats (male, 250 ± 50 g), Marmarou weight-drop TBI model	*n* = 8/group	10, 25, 50 mg/kg i.p.	1 week	Ketamine + xylazine (80 + 15 mg/kg)	I (PA); II (HE, Nissl, Dark neuron quantification); III (MDA, SOD, CAT) IV (−); V (−)
Fu et al. (16)	[Cognitive impairment] SD rats (male, 250–300 g), 2-VO chronic cerebral hypoperfusion	*n* = 20/group	50, 100, 200 mg/kg p.o.	8 weeks	10% chloral hydrate (350 mg/kg)	I (MWM); II (−); III (SOD, MDA, GSH); IV (TNF-α, IL-1β, NF-*κ*B); V (Aβ1-40/42, BACE1)
He et al. (17)	[Cognitive impairment] SD rats (male, 8-week-old, 280–300 g), 2-VO chronic cerebral hypoperfusion	*n* = 15/group	50, 100 mg/kg i.g.	3 months	NR	I (MWM, ORT); II (−); III (−); IV (−); V (PI3K-p110α, PI3K-p85, Akt, P-Akt)
He et al. (18)	[AD-specific] 3 × Tg-AD mice (female, 8-month-old)	*n* = 12/group	20, 40 mg/kg i.p.	8 weeks	NR (isoflurane for surgery)	I (NOR, Step-down Avoidance, OFT); II (TEM, IF, TUNEL); III (SOD, MDA, GSH, ROS, MMP, OCR/ECAR); IV (−); V (PPAR*γ*, BACE1, IDE, Aβ, CytC, Caspase-3/9)
Jang et al. (19)	[Cognitive impairment] Balb/c mice (male, adult 3–6 month; aged 22–24 month)	*n* = 12–14/group	∼19.9 mg/day (0.6% luteolin in AIN-93M diet)	4 weeks	CO₂ asphyxiation	I (MWM); II (−); III (PGE₂, Nitrite); IV (IL-1β, TNF-α, IL-6, MHC II, iNOS, COX-2); V (−)
Kou et al. (20)	[AD-specific] 3 × Tg-AD mice (8-month-old, male/female)	*n* = 8/group	20, 40 mg/kg i.p.	3 weeks	NR	I (MWM); II (Nissl, Thioflavin S, IF: GFAP/GRP78); III (−); IV (GFAP, TNF-α, IL-1β, IL-6, NO, COX-2, iNOS); V (ER Stress: GRP78, IRE1α, p-NF-κB, p-p38)
Li et al. (21)	[Cognitive impairment] Mice, D-galactose subcutaneous injection (120 mg/kg) aging model	*n* = 10/group	120 mg/kg i.g.	NR	Chloral hydrate (40 mg/kg)	I (MWM, NOR); II (IF: NeuN); III (SOD, MDA); IV (−); V (SYN, PSD-95)
Li et al. (22)	[AD-specific] SD rats (male, SPF), Aβ25–35 (5 μg) bilateral hippocampal injection + D-galactose (100 mg/kg, 42 days)	*n* = 12/group	25, 50, 100 mg/kg i.p.	14 days	Pentobarbital sodium	I (NOR); II (HE, TUNEL); III (SOD, GSH); IV (IL-1β, IL-10, IL-6); V (SDF-1α, CXCR4)
Liu et al. (23)	[AD-specific] SD rats (male, 6-week-old, 190–240 g), Aβ25–35 bilateral hippocampal CA1 injection (4 μg/μL)	*n* = 12/group	20, 40 mg/kg i.g.	NR	NR	I (MWM); II (HE, TUNEL); III (SOD, MDA); IV (TNF-α, IL-6); V (NGF, TrkA)
Park et al. (24)	[AD-specific] SD rats (male, 202 ± 11 g), Aβ(25–35) infusion + HFD	*n* = 20/group	∼50 mg/kg diet (0.05% luteolin + 0.1% L-theanine)	42 days	Ketamine + xylazine (100 + 10 mg/kg)	I (MWM, PA); II (−); III (−); IV (TNF-α, IL-1β, IL-6); V (pAkt/Akt, pGSK-3β, pFOXO-1, pCREB, pTau, Norepinephrine, OGTT/HOMA-IR)
Sawmiller et al. (25)	[AD-specific] Tg2576 mice (6Female/6Male), controlled cortical impact TBI	*n* = 12/group	20 mg/kg i.p.	15 days	Isoflurane	I (−); II (Thionin staining); III (−); IV (TNF-α, IL-1β); V (Aβ40/42, p-GSK-3α/β, GSK-3, p-tau, tau)
Tao et al. (26)	[AD-specific] C57BL/6J mice (male, 7–8 weeks), Aβ1–42 oligomers bilateral hippocampal CA1 injection	*n* = 12/group	100 mg/kg i.g. + voluntary wheel running	4 weeks	2% isoflurane	I (MWM); II (IHC: DCX, NeuN); III (−); IV (−); V (LC3, autophagy, metabolomics: purine/retinol/thiamine/histidine/cysteine-methionine pathways)
Yu et al. (27)	[AD-specific] SD rats (male), ICV Aβ(1–40) (2 μL, 1 mM/μL)	*n* = 10/group	50, 100, 200 mg/kg p.o.	17 days	10% chloral hydrate (300 mg/kg)	I (MWM, PA); II (−); III (SOD, GSH-Px, MDA); IV (−); V (ACh, AchE, ChAT, Bcl-2/Bax)
Zhang et al. (28)	[Cognitive impairment] SD rats (male, 200–220 g), Scopolamine i.p. (1 mg/kg)	*n* = 10/group	30, 60, 120 mg/kg i.p.	28 days	NR	I (MWM, PA); II (−); III (SOD, MDA); IV (−); V (AchE)
Zhou et al. (29)	[Cognitive impairment] C57 mice (8–9 weeks, 20–25 g, half male/female), LPS i.p. (250 μg/kg/day)	*n* = 8/group	120 mg/kg i.g.	14 days	NR	I (MWM); II (Nissl); III (NO); IV (TNF-α, IL-1β); V (−)

Data are presented as reported in each original study. I. Behavioral/cognitive tests—MWM, NOR, PA, Step-down avoidance, Y-maze, OFT, FST, Barnes maze, ORT; II. Histopathological assessment—HE, Nissl, Thioflavin S/Congo red, TUNEL assay, TEM, IHC/IF, NeuN, DCX, MAP-2, Thionin, dark neuron quantification, nanocarrier characterization, BBB permeability, 16S rRNA sequencing, FMT; III. Oxidative stress and mitochondrial function—SOD, CAT, GSH, GSH-Px, MDA, ROS, NO, MMP, UCP2, PGC-1*α*, NRF1/2, TFAM, Drp1, Fis1, Mfn1/2, OCR/ECAR, PGE₂, Nitrite; IV. Neuroinflammation—TNF-α, IL-1β, IL-6, IL-10, TGF-*β*, iNOS, COX-2, NF-*κ*B, GFAP, IBA1, CD11b, MHC II, MCP-1; V. Signaling pathways and other targets.

### 3.3 Quality assessment and risk of bias

The SYRCLE tool for assessing the risk of bias in animal studies was used to evaluate the methodological quality of the 15 included preclinical studies in rodents. Figure 2A summarizes the specific assessment results for each domain in each study, while Figure 2B shows the proportion distribution of risk ratings for various biased domains. Only one study was rated as having a low risk for random sequence generation; the remainder were rated as having an unknown risk. All included studies received a low-risk rating for baseline characteristics. Due to a lack of methodological descriptions, all studies were rated as “unknown risk” for allocation concealment, blinding of intervention personnel, blinded outcome assessment, and other sources of bias. One study reported randomization, and another implemented blinded outcome assessment; both of these studies were rated as low risk. Three studies adequately documented animal dropouts and sample exclusion criteria and were rated as low risk; the remaining studies were classified as “unknown risk” due to incomplete outcome data. Overall, although some studies did not report key details such as animal age, sex, or allocation concealment, most studies provided comprehensive and satisfactory reporting in other respects. The “unknown risk” rating predominated in the included literature, primarily due to incomplete methodological reporting in the original studies rather than identified actual experimental flaws.

**Figure 2:**
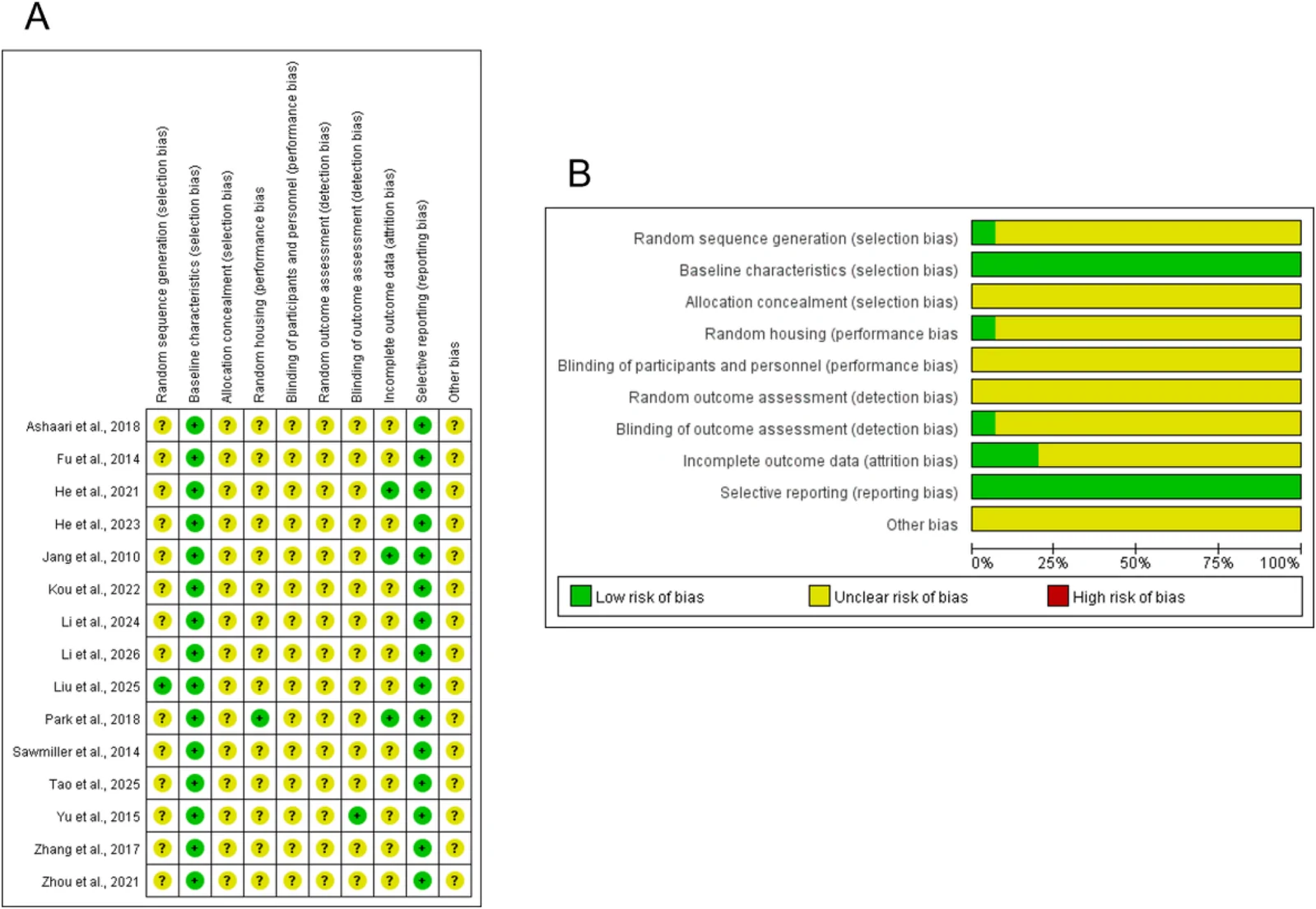
Methodological quality and risk of bias assessment. **(A)** Summary of risk of bias for each included study. **(B)** Proportional distribution of risk of bias domains across all studies.

### 3.4 Meta analysis

#### 3.4.1 Morris water maze escape latency

This study analyzed escape latency in the Morris Water Maze. The overall meta-analysis results indicated that luteolin intervention significantly reduced escape latency in animals with AD and cognitive impairment. Given the high heterogeneity among the 13 datasets derived from 9 independent studies (*P* < 0.0001, *I*^2^ = 86.6%), a random-effects model was used for the meta-analysis. The overall effect size showed that the escape latency in the luteolin treatment group was significantly lower than that in the model control group (SMD = −3.59, 95% CI: [−4.92, −2.26], *Z* = 5.28, *P* < 0.0001), which indicates that this natural flavonoid exerts a significant reversing and protective effect against spatial cognitive decline.

To explicitly address the etiological heterogeneity among the experimental paradigms and evaluate model relevance, a predefined subgroup analysis was conducted stratifying the studies into AD-specific models and cognitive impairment models. The pooled results demonstrated that luteolin significantly reduced escape latency in both AD-specific models (SMD = −3.35, 95% CI: [−5.19, −1.50]) and cognitive impairment models (SMD = −3.91, 95% CI: [−6.03, −1.80]). The test for subgroup differences revealed no statistical significance (*P* = 0.6932), indicating that the therapeutic efficacy of luteolin in improving spatial navigation is consistent across both core AD-defining pathologies and broader etiological triggers of cognitive decline.

To investigate the potential sources of the high heterogeneity observed in the overall results, a predefined subgroup analysis was conducted based on “duration of intervention”. In the stratified comparison (≤4 weeks vs. >4 weeks), the subgroup difference test reached statistical significance (*P* = 0.0108). Specifically, long-term interventions (>4 weeks) demonstrated a more pronounced effect on escape latency reduction (SMD = −5.01, 95% CI: [−7.38, −2.64]). Notably, in the short-term intervention (≤4 weeks) subgroup, heterogeneity among studies was completely eliminated (*I*^2^ = 0%), and the effect remained highly statistically significant (SMD = −1.86, 95% CI: [−2.35, −1.37]). This finding identifies intervention duration as a key source of overall heterogeneity, with larger effect sizes observed in studies utilizing interventions longer than 4 weeks. However, due to confounding across different studies, models, and doses, this does not necessarily establish a time-dependent cumulative pharmacological effect (Figure 3).

**Figure 3:**
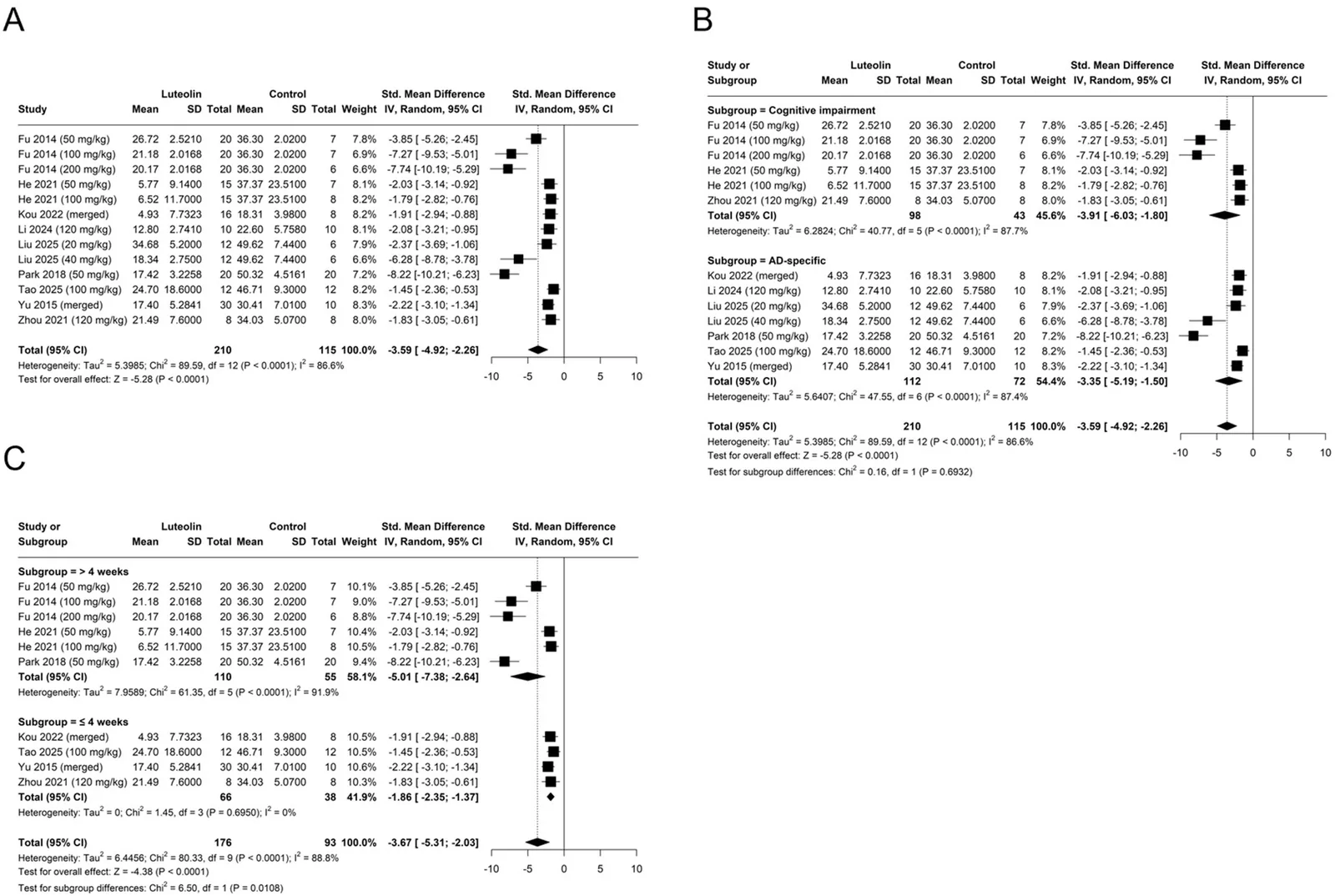
Forest plot of the effect of luteolin on Morris water maze escape latency and subgroup analyses by intervention duration and animal model.

Furthermore, since the vast majority of the included studies (12 studies) used oral administration and only one study used intraperitoneal injection, no formal subgroup analysis was conducted for “route of administration”. This decision was made to avoid insufficient statistical power due to extreme imbalance in sample sizes between groups.

#### 3.4.2 Morris water maze target quadrant time

Target quadrant time is a core behavioral indicator for evaluating spatial memory retention and consolidation in animal models. The overall meta-analysis demonstrated that the time spent in the target quadrant by the luteolin-treated group was significantly longer than that of the model control group. Given the substantial heterogeneity among the 10 datasets derived from 7 independent studies (*P* < 0.0001, *I*^2^ = 82.8%), a random-effects model was employed for the pooled analysis. The overall effect size indicated that luteolin significantly reversed memory retention deficits in the model animals (SMD = 2.68, 95% CI: [1.72, 3.64], *Z* = 5.48, *P* < 0.0001).

To explore the potential sources of this high heterogeneity, multidimensional predefined subgroup analyses were conducted. First, to explicitly address the etiological diversity among the experimental paradigms, a subgroup analysis was performed stratifying the studies into AD-specific models and cognitive impairment models. The pooled results demonstrated that luteolin significantly prolonged the target quadrant time in both AD-specific models (SMD = 2.06, 95% CI: [1.32, 2.80]) and cognitive impairment models (SMD = 3.06, 95% CI: [1.53, 4.59]). The test for subgroup differences revealed no statistical significance (*P* = 0.2472), indicating that luteolin's efficacy in enhancing spatial memory retention is remarkably consistent across core AD-defining pathologies and broader distinct triggers of cognitive decline.

Subsequently, another stratified comparison based on the “route of administration” reached statistical significance (*P* = 0.0074). Specifically, the oral administration subgroup exhibited a significant and stable efficacy in memory improvement (SMD = 3.11, 95% CI: [2.09, 4.12]); meanwhile, the intraperitoneal administration subgroup, despite a relatively smaller pooled effect size, also demonstrated a significant protective effect (SMD = 1.12, 95% CI: [0.08, 2.16]). These results clearly suggest that luteolin can achieve significant central neuroprotection and cognitive improvement via a non-invasive, conventional oral route. These results clearly suggest that luteolin can achieve significant central neuroprotection and cognitive improvement via a non-invasive, conventional oral route. However, due to observational confounding across distinct experimental designs, these subgroup differences should not be misinterpreted as definitive evidence of the pharmacokinetic superiority of oral delivery (Figure 4).

**Figure 4:**
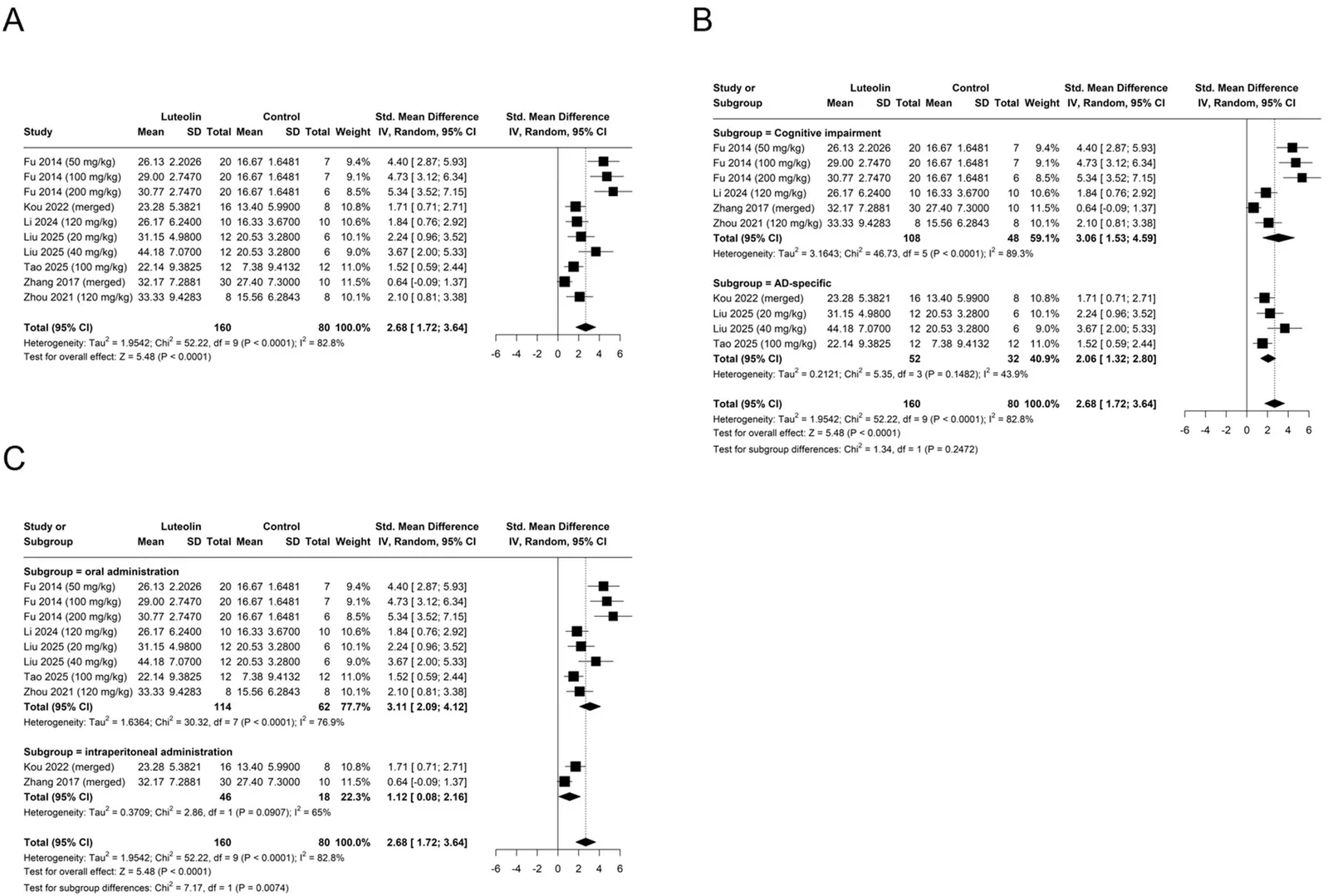
Meta-analysis and subgroup analyses of luteolin on target quadrant time in the Morris water maze.

Taken together, these subgroup analyses demonstrate that the route of administration is a key source of heterogeneity for this indicator, whereas the underlying disease model etiology does not significantly confound luteolin's efficacy in spatial memory consolidation.

#### 3.4.3 Morris water maze platform crossings

The number of platform crossings is a crucial behavioral indicator for evaluating the accurate memory retrieval capacity of spatial locations in animal models. The overall meta-analysis revealed that luteolin intervention significantly increased the number of platform crossings in animals with AD and cognitive impairment. Given the substantial heterogeneity among the 8 datasets derived from 5 independent studies (*P* < 0.0001, *I*^2^ = 87.2%), a random-effects model was employed for the pooled analysis. The overall effect size confirmed that luteolin exerts a significant restorative effect on impaired accurate spatial memory (SMD = 2.65, 95% CI: [1.37, 3.93], *Z* = 4.07, *P* < 0.0001).

To further investigate the sources of high heterogeneity and the differences in efficacy among various intervention strategies, multidimensional predefined subgroup analyses were conducted. First, to explicitly evaluate the impact of etiological diversity, studies were stratified into AD-specific models and cognitive impairment models. The pooled results demonstrated that luteolin significantly increased the number of platform crossings in both AD-specific models (SMD = 2.55, 95% CI: [1.14, 3.97]) and cognitive impairment models (SMD = 2.71, 95% CI: [0.72, 4.71]). The test for subgroup differences revealed no statistical significance (*P* = 0.8983), indicating that luteolin's restorative effect on accurate spatial memory retrieval is highly consistent across core AD-defining pathologies and broader etiological triggers of cognitive decline.

Subsequently, in the stratified comparison based on the “route of administration” the test for subgroup differences showed high statistical significance (*P* = 0.0093). It is particularly noteworthy that the oral administration subgroup exhibited robust and definitive efficacy in memory improvement (SMD = 3.27, 95% CI: [1.86, 4.68]). In contrast, although the pooled effect size of the intraperitoneal administration subgroup showed a positive trend, its 95% confidence interval crossed the line of no effect, failing to reach statistical significance (SMD = 0.92, 95% CI: [−0.15, 1.99]). This finding not only identifies the route of administration as a core source of heterogeneity for this indicator but also indicates that orally administered luteolin is associated with significant improvements in accurate spatial navigational memory. However, this observational finding should be interpreted cautiously due to potential confounding factors across studies (Figure 5).

**Figure 5:**
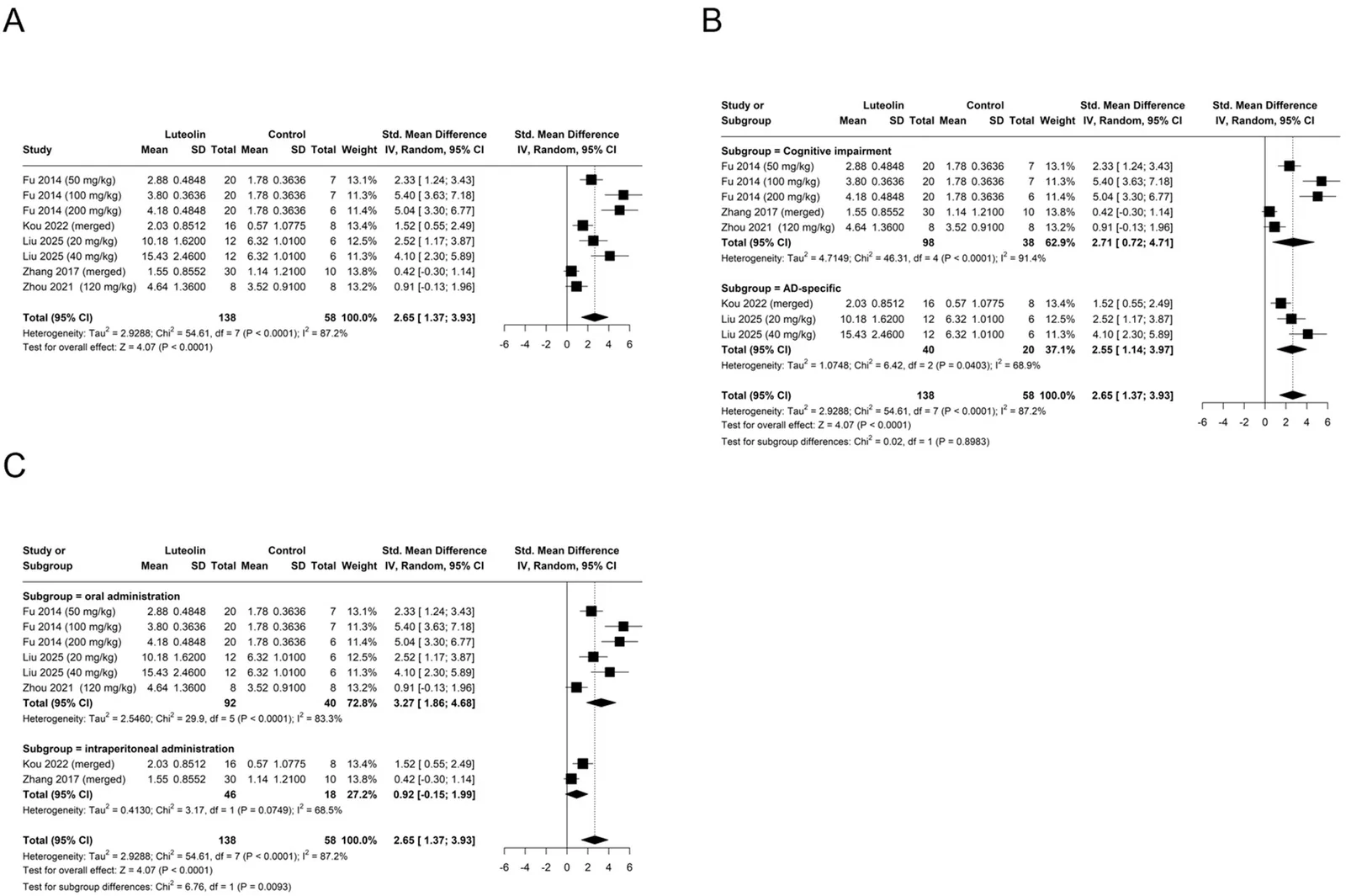
Meta-analysis and subgroup analyses of luteolin on platform crossings in the Morris water maze.

Taken together, these subgroup analyses demonstrate that the route of administration is a key source of heterogeneity for this indicator, whereas the underlying disease model etiology does not significantly confound luteolin's efficacy in spatial memory retrieval capacity.

#### 3.4.4 Passive avoidance step-through latency

The step latency in the passive avoidance (PA) test is a key measure for evaluating associative learning and passive avoidance memory in rodents. An overall meta-analysis indicated that, compared with the model control group, the step latency was significantly prolonged in the luteolin-treated group. Given the significant heterogeneity among the 6 datasets derived from 5 independent studies (*P* < 0.0001, *I*^2^ = 82.3%), a random-effects model was employed. The overall meta-analysis confirmed that luteolin effectively reversed avoidance memory deficits in model animals (SMD = 1.81, 95% CI: [0.78, 2.85], *Z* = 3.43, *P* = 0.0006).

To investigate the source of the high heterogeneity, multidimensional predefined subgroup analyses were conducted. First, to rigorously evaluate the impact of etiological diversity, studies were stratified into AD-specific models and cognitive impairment models. The pooled results demonstrated that luteolin significantly prolonged the step-through latency in both AD-specific models (SMD = 2.10, 95% CI: [0.53, 3.67]) and cognitive impairment models (SMD = 1.25, 95% CI: [0.52, 1.98]). The test for subgroup differences revealed no statistical significance (*P* = 0.3360), indicating that the protective effect of luteolin on associative learning and passive avoidance memory is consistent across core AD-defining pathologies and broader etiological triggers of cognitive decline.

Subsequently, in the stratified analysis based on “route of administration” heterogeneity was completely eliminated within the intraperitoneal administration subgroup (*I*^2^ = 0%), indicating a highly stable and well-defined memory-protective effect (SMD = 1.13, 95% CI: [0.66, 1.60]). In contrast, the oral administration subgroup exhibited a larger mean effect size (SMD = 3.22, 95% CI: [0.93, 5.50]), but with higher heterogeneity. The subgroup heterogeneity test showed a trend toward significance (*P* = 0.0797). This finding not only perfectly explains the primary source of overall heterogeneity but also suggests that non-oral administration may have a potential advantage in maintaining pharmacological stability.

Furthermore, in the subgroup analysis based on “intervention duration” (≤4 weeks vs. >4 weeks), no statistically significant differences were observed between the groups (*P* = 0.6337). Short-term interventions (≤4 weeks) demonstrated significant and consistent efficacy (SMD = 1.53, 95% CI: [0.83, 2.23]). However, in the long-term intervention (>4 weeks) subgroup, confounding factors resulting from different routes of administration across studies led to a sharp increase in heterogeneity within the subgroup (*I*^2^ = 91%), causing the pooled effect size to lose statistical significance (95% CI: [−0.16, 4.36]) (Figure 6). A comprehensive comparison of these results indicates that, compared to intervention duration, the route of administration is the key covariate responsible for the observed differences in the efficacy of luteolin on passive avoidance memory.

**Figure 6:**
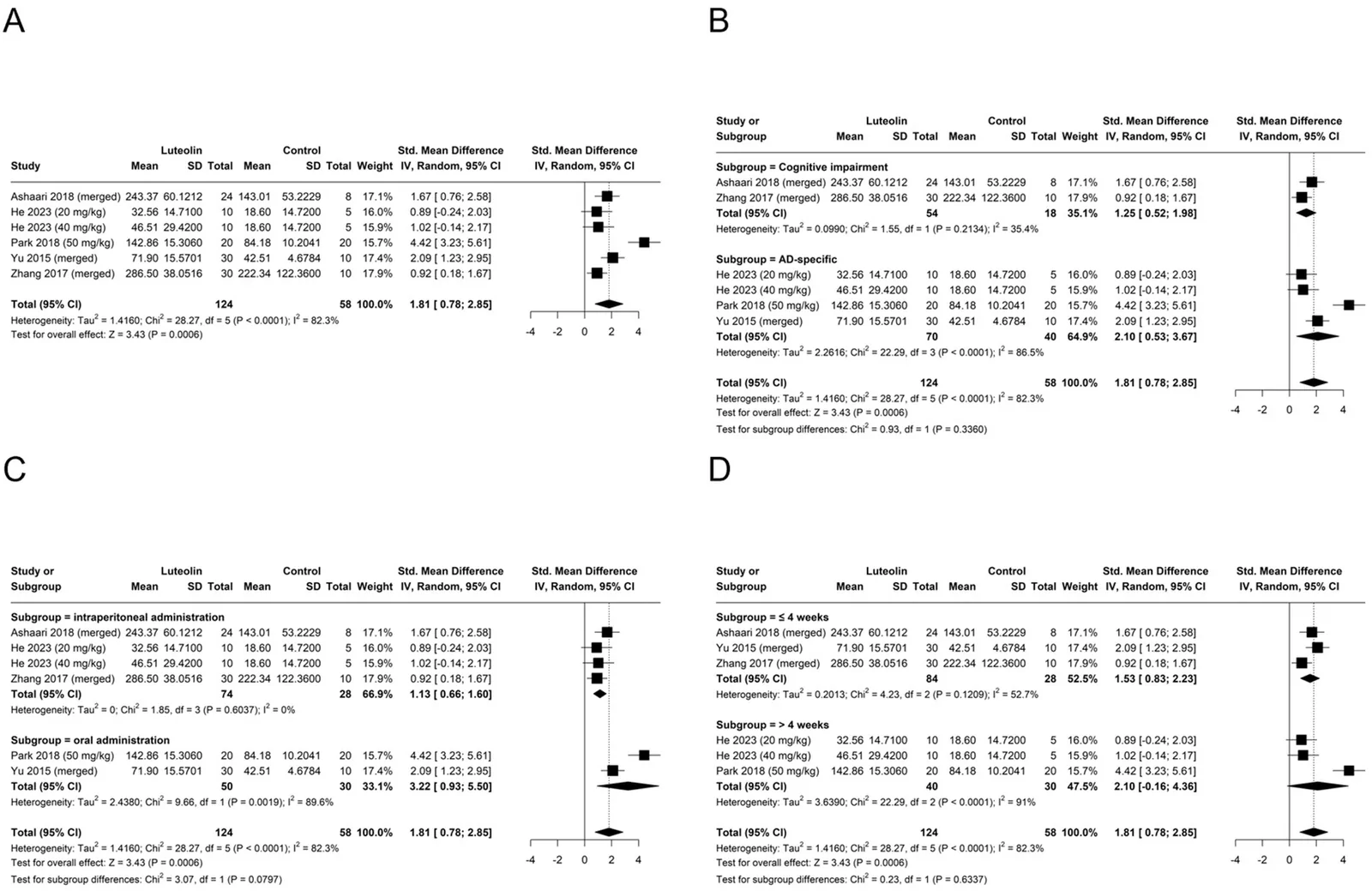
Meta-analysis and subgroup analyses of luteolin on step-through latency in the passive avoidance test.

#### 3.4.5 Novel object recognition index

The recognition index in the novel object recognition (NOR) test is a core indicator for evaluating non-spatial recognition memory in rodents. The overall meta-analysis revealed that the recognition index of the luteolin-treated group was significantly higher than that of the model control group. Given the moderate heterogeneity among the 5 datasets derived from 3 independent studies (*P* = 0.0104, *I*^2^ = 69.6%), a random-effects model was adopted. The pooled effect size confirmed that luteolin could effectively reverse recognition memory impairment in model animals (SMD = 1.72, 95% CI: [0.89, 2.55], *Z* = 4.08, *P* < 0.0001) (Figure 7).

**Figure 7:**
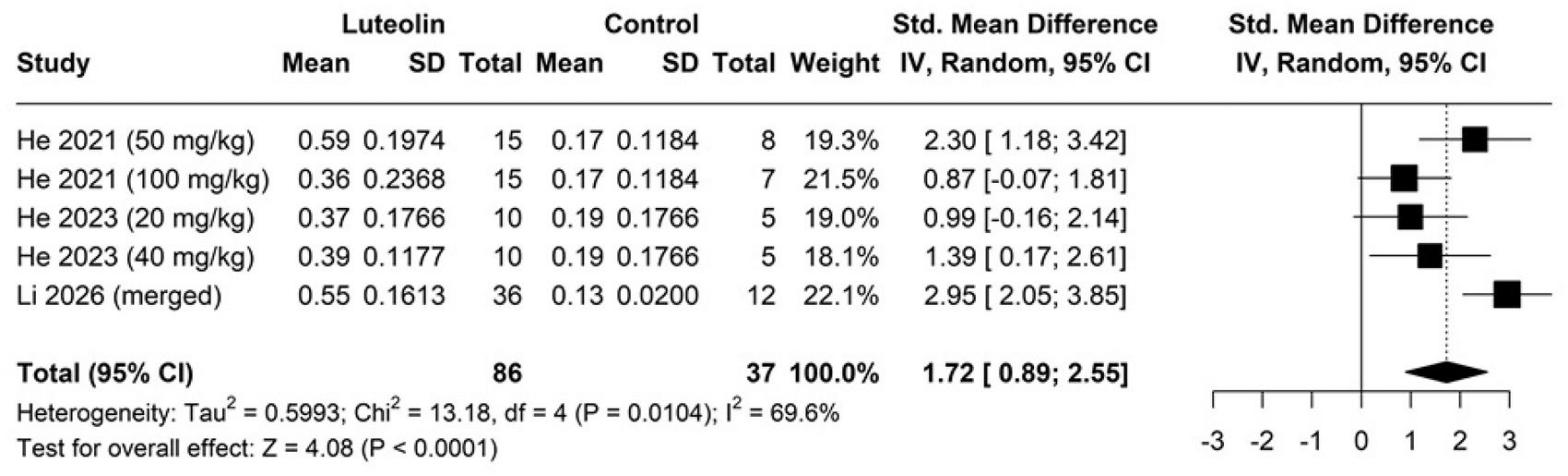
Meta-analysis of luteolin on the novel object recognition index.

Furthermore, predefined subgroup analyses based on potential covariates (such as disease models, route of administration, and intervention duration) were initially planned. However, due to the limited number of included datasets and the resulting insufficient statistical power, quantitative subgroup partitioning was not performed to avoid single-source confounding bias and unreliable estimations.

#### 3.4.6 Oxidative stress biomarker (SOD) levels

SOD is a core antioxidant enzyme responsible for scavenging free radicals and defending against oxidative stress injury *in vivo*. The overall meta-analysis revealed that luteolin intervention remarkably elevated SOD activity levels in model animals. Given the high heterogeneity among the 10 datasets derived from 8 independent studies (*P* < 0.0001, *I*^2^ = 76.7%), a random-effects model was employed for pooled calculation. The overall effect size confirmed that luteolin exerts a significantly antioxidant reversing effect against central or systemic oxidative stress damage (SMD = 2.55, 95% CI: [1.77, 3.33], *Z* = 6.40, *P* < 0.0001).

First, to explicitly address the etiological heterogeneity and assess the impact of diverse disease models, a predefined subgroup analysis was conducted stratifying the datasets into AD-specific models and cognitive impairment models. The pooled results demonstrated that luteolin significantly upregulated SOD activity in both AD-specific models (SMD = 2.12, 95% CI: [1.63, 2.61]) and cognitive impairment models (SMD = 2.95, 95% CI: [1.61, 4.29]). The test for subgroup differences revealed no statistical significance (*P* = 0.2555), indicating that luteolin's capacity to restore endogenous antioxidant defenses is consistent across both amyloid-driven pathologies and broader neuroinflammatory or aging-related models of cognitive decline. Notably, heterogeneity was substantially reduced within the AD-specific subgroup (*I*^2^ = 3.5%), suggesting that the etiological difference among animal models is a contributing factor to the overall variance.

Subsequently, in the stratified comparison based on the “route of administration” the test for subgroup differences reached statistical significance (*P* < 0.0001). Notably, the between-study heterogeneity in the intraperitoneal administration subgroup was completely eliminated (*I*^2^ = 0%), demonstrating a highly stable SOD-elevating effect (SMD = 1.47, 95% CI: [1.01, 1.93]). Concurrently, the heterogeneity in the oral administration subgroup was substantially reduced (*I*^2^ = 52.7%), presenting a significant antioxidant enhancing effect (SMD = 3.34, 95% CI: [2.56, 4.13]). This finding identifies the route of administration as a major source of overall heterogeneity and confirms that the oral route is associated with significant efficacy in activating the endogenous antioxidant defense system. Nevertheless, pharmacokinetic properties cannot be directly inferred from these biochemical effect sizes.

Furthermore, a second subgroup analysis was conducted using “intervention duration” as a covariate (≤4 weeks vs. >4 weeks). To strictly adhere to the data rigor of meta-analysis, the two datasets corresponding to Li 2024 (21) and Liu 2025 (23) were excluded in this section, as their original texts did not provide explicit information regarding the administration duration. The test based on the remaining eight datasets revealed a distinct trend of marginal statistical significance between the groups (*P* = 0.0639). Short-term intervention (≤4 weeks) was already capable of significantly upregulating SOD activity (SMD = 1.75, 95% CI: [1.06, 2.44]), whereas long-term intervention (>4 weeks) exhibited a substantially stronger effect size (SMD = 3.48, 95% CI: [1.79, 5.18]) (Figure 8). This trend not only corroborates that the intervention duration is a key covariate affecting efficacy variance but also highlights that effect sizes differed between studies categorized by intervention duration. However, this observational difference cannot conclusively establish that prolonging treatment inherently enhances efficacy.

**Figure 8:**
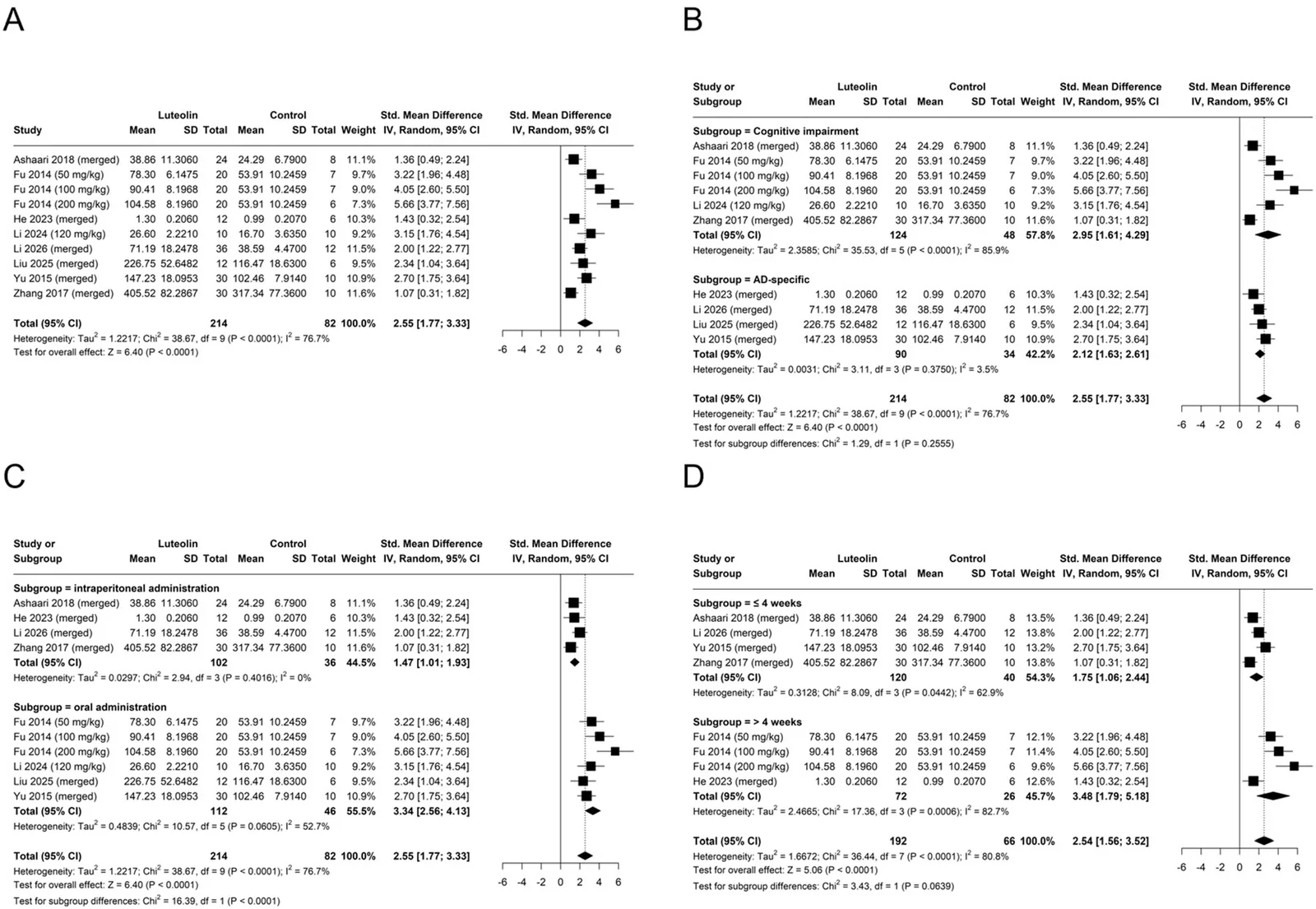
Meta-analysis and subgroup analyses of luteolin on SOD activity.

#### 3.4.7 Core endogenous antioxidant (GSH) levels

GSH is a crucial endogenous antioxidant essential for defending against oxidative stress injury and maintaining cellular redox homeostasis. The overall meta-analysis revealed that luteolin intervention remarkably elevated GSH levels in model animals. Given the high heterogeneity among the 5 datasets derived from 3 independent studies (*P* < 0.0001, *I*^2^ = 88.5%), a random-effects model was employed for the pooled analysis. The overall effect size confirmed that luteolin possesses significant capacity to reverse antioxidant depletion (SMD = 3.51, 95% CI: [1.62, 5.41], *Z* = 3.64, *P* = 0.0003) (Figure 9).

**Figure 9:**
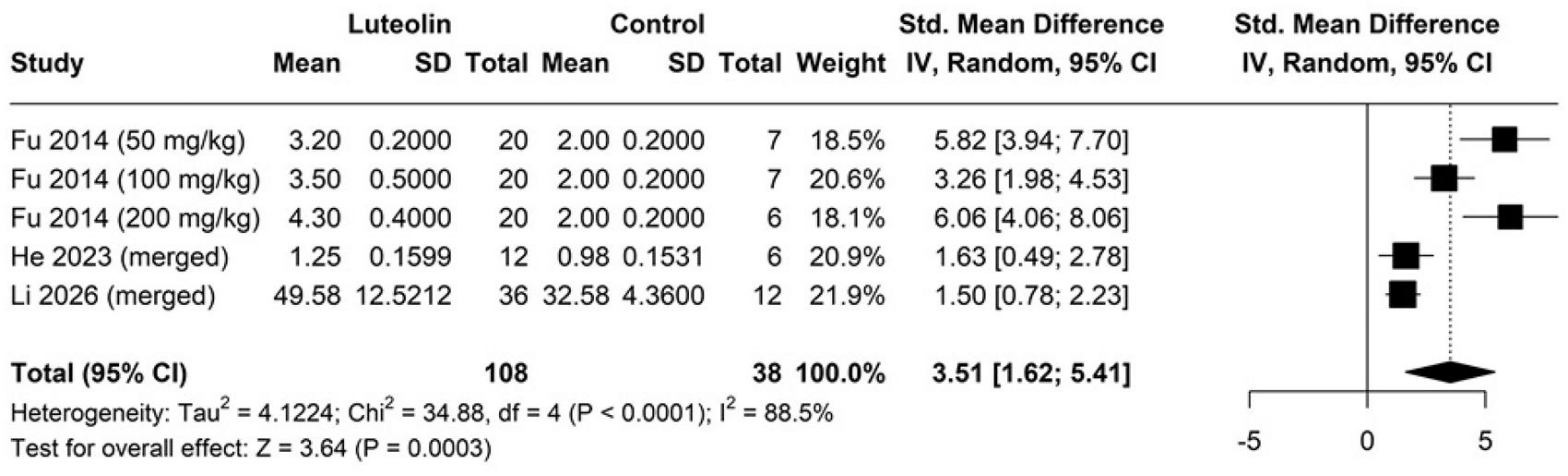
Meta-analysis of luteolin on GSH levels.

Although additional stratified evaluations—such as examining different etiological models, delivery methods, or treatment lengths—were initially considered, they were ultimately omitted. The sparse pool of available studies yielded inadequate statistical strength for reliable subgroup comparisons; therefore, bypassing these breakdowns prevents skewed estimates and single-source distortion.

#### 3.4.8 Lipid peroxidation biomarker (MDA) levels

MDA, as the end product of lipid peroxidation, is a hallmark biochemical indicator for assessing the severity of oxidative stress injury *in vivo*. The overall meta-analysis revealed that luteolin intervention remarkably reduced MDA content in model animals. Given the high heterogeneity among the 9 datasets derived from 7 independent studies (*P* < 0.0001, *I*^2^ = 91.2%), a random-effects model was employed for the pooled analysis. The overall effect size demonstrated that luteolin possesses a significant inhibitory effect on lipid peroxidation damage (SMD = −3.01, 95% CI: [−4.40,−1.62], *Z* = 4.23, *P* < 0.0001).

First, to rigorously evaluate the impact of etiological diversity, a predefined subgroup analysis was conducted stratifying the datasets into AD-specific models and cognitive impairment models. The pooled results demonstrated that luteolin significantly reduced MDA levels in both AD-specific models (SMD = −1.92, 95% CI: [−2.97,−0.86]) and cognitive impairment models (SMD = −3.56, 95% CI: [−5.53, −1.58]). The test for subgroup differences revealed no statistical significance (*P* = 0.1508), indicating that luteolin's efficacy in mitigating lipid peroxidation is remarkably consistent across both core AD-defining pathologies and broader etiological triggers of cognitive decline. Additionally, within-subgroup heterogeneity was reduced in the AD-specific category (*I*^2^ = 63%), suggesting that etiological differences partially account for the overall variance.

Subsequently, to deeply explore additional sources of the high heterogeneity, multidimensional predefined subgroup analyses were conducted. In the stratified comparison based on the “route of administration” the test for subgroup differences reached a highly significant statistical level (*P* = 0.0091). It is particularly noteworthy that the between-study heterogeneity in the intraperitoneal administration subgroup was completely eliminated (*I*^2^ = 0%), demonstrating a highly stable and definitive MDA-clearing effect (SMD = −1.23, 95% CI: [−1.90, −0.56]). Concurrently, the oral administration subgroup, despite retaining some heterogeneity, exhibited a substantially more powerful overall effect size (SMD = −3.55, 95% CI: [−5.16, −1.94]). This finding identifies the route of administration as a core source of the overall heterogeneity and confirms the significant *in vivo* efficacy of orally administered luteolin in mitigating lipid peroxidation, though translational advantages require further controlled pharmacological validation.

Furthermore, a subgroup analysis was performed using “intervention duration” (≤4 weeks vs. >4 weeks) as a covariate. To ensure the rigor of the systematic review and the certainty of the data, two datasets corresponding to Li 2024 (21) and Liu 2025 (23) were excluded in this section, as their original texts did not explicitly report the administration duration. The test based on the remaining seven datasets revealed a significant difference between the groups (*P* = 0.0274). Short-term intervention (≤4 weeks) was already capable of effectively reducing MDA levels (SMD = −1.41, 95% CI: [−2.23, −0.59]), whereas long-term intervention (>4 weeks) demonstrated a substantially more prominent pharmacological effect (SMD = −4.46, 95% CI: [−7.04,−1.87]). This result indicates that larger effect sizes were observed in the long-term intervention subgroup. Nevertheless, due to study-level confounding, this does not establish a definitive time-dependent cumulative characteristic of its efficacy (Figure 10).

**Figure 10:**
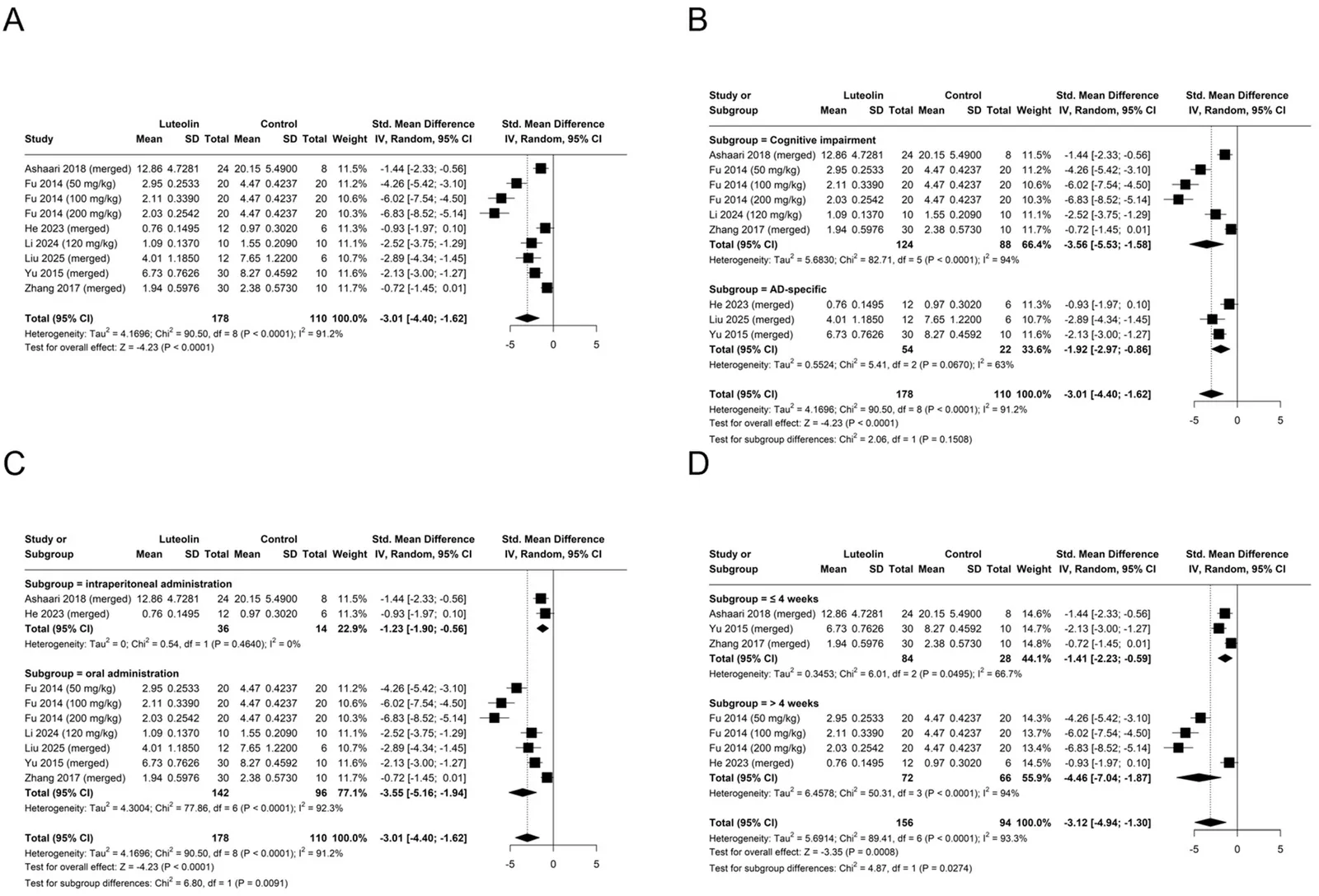
Meta-analysis and subgroup analyses of luteolin on MDA levels.

#### 3.4.9 Neuroinflammatory cytokine expression at transcriptional and translational levels

The abnormal neuroinflammatory cascade is a pivotal pathological mechanism driving the exacerbation of cognitive impairment. To comprehensively evaluate the anti-inflammatory mechanism of luteolin, this study systematically reviewed core pro-inflammatory cytokines (IL-1β, IL-6, TNF-α) from the dual dimensions of gene transcription and protein expression.

At the transcriptional level, the meta-analysis results demonstrated that luteolin intervention significantly suppressed the mRNA transcription of pro-inflammatory cytokines in the hippocampus or cortex of model animals. Specifically, luteolin significantly down-regulated the expression levels of TNF-α mRNA (SMD = −2.74, 95% CI: [−4.20, −1.28], *Z* = 3.68, *P* = 0.0002) and IL-1β mRNA (SMD = −9.10, 95% CI: [−15.70, −2.50], *Z* = 2.70, *P* = 0.0069) (Figure 11). This finding confirms its anti-inflammatory potential fundamentally from the source of gene transcription.

**Figure 11:**
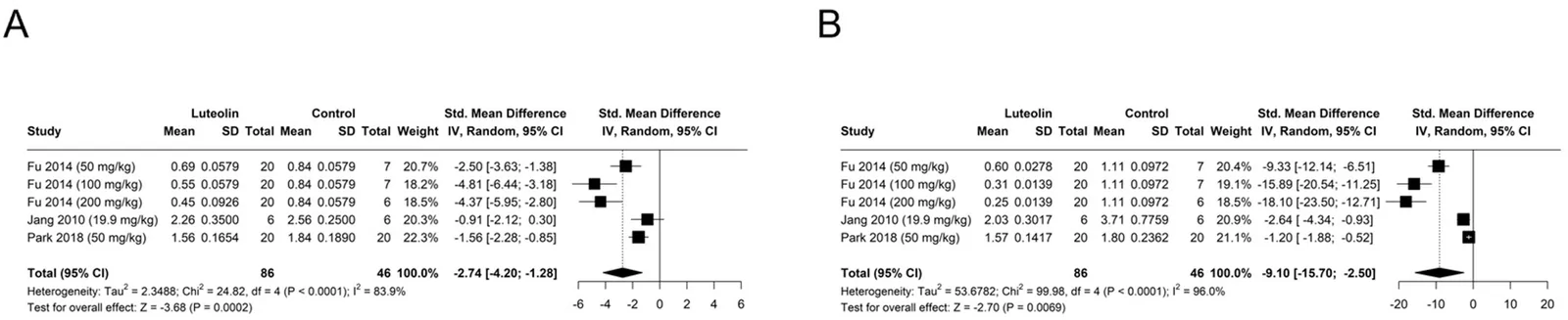
Meta-analysis of the effect of luteolin on TNF-α and IL-1β mRNA expression levels. **(A)** Meta-analysis of TNF-α mRNA expression levels. **(B)** Meta-analysis of IL-1β mRNA expression levels.

At the translational and secretory level, the overall pooled results of ELISA data further corroborated the aforementioned findings. Luteolin significantly reduced the excessive protein secretion of IL-1β (SMD = −9.77, 95% CI: [−16.05, −3.49], *P* = 0.0023) (Figure 12), IL-6 (SMD = −3.08, 95% CI: [−3.84, −2.33], *P* < 0.0001) (Figure 13), and TNF-α (SMD = −8.06, 95% CI: [−11.67,−4.46], *P* < 0.0001) (Figure 14). Among these, the pooled results for IL-6 exhibited low heterogeneity (*I*^2^ = 37.3%), indicating a high degree of consistency across studies for this anti-inflammatory target. Conversely, considerable heterogeneity was observed in the protein levels of IL-1β (*I*^2^ = 94.1%) and TNF-α (*I*^2^ = 92.5%).

**Figure 12:**
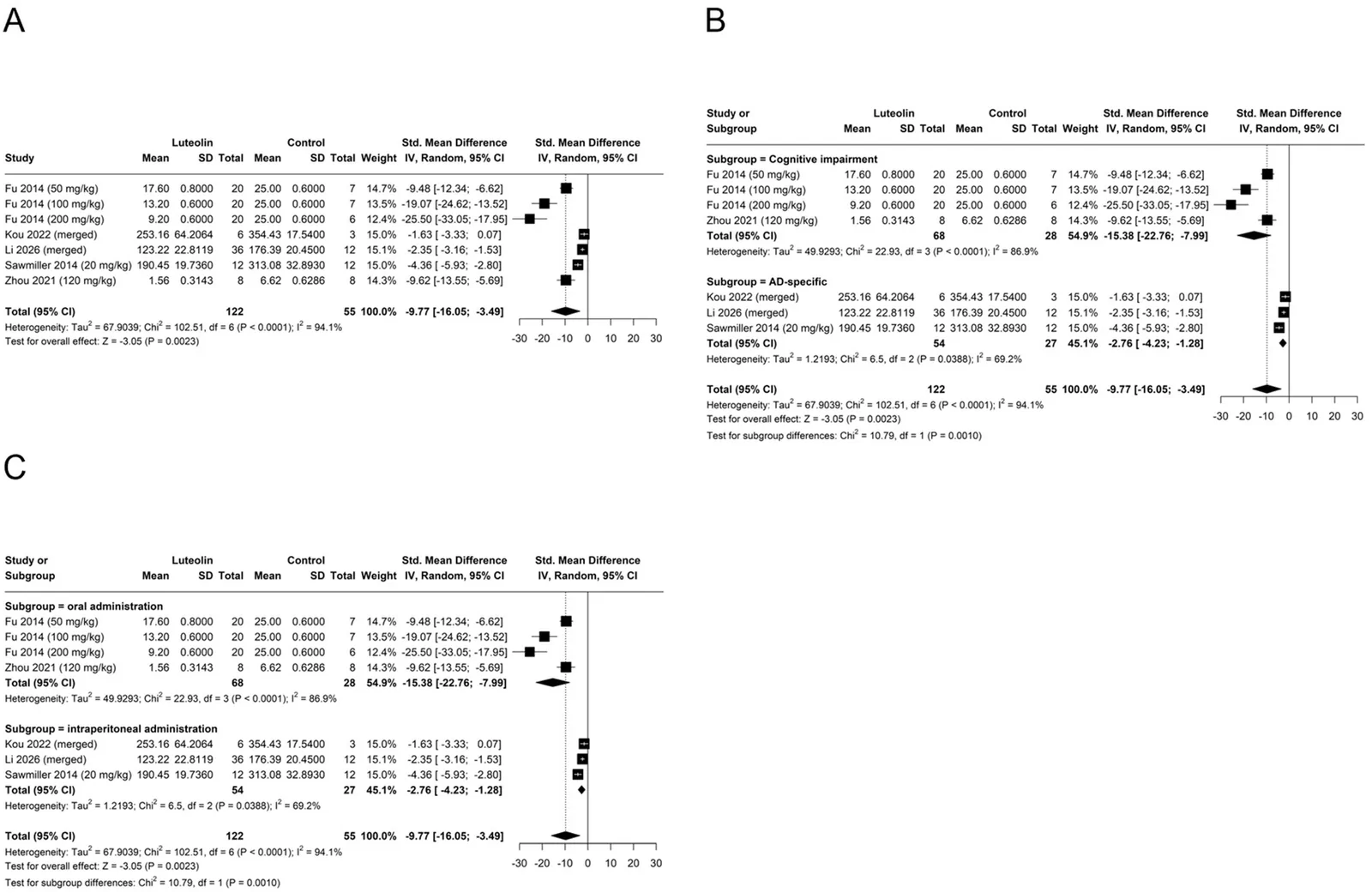
Meta-analysis and subgroup analyses of luteolin on IL-1β protein levels.

**Figure 13:**
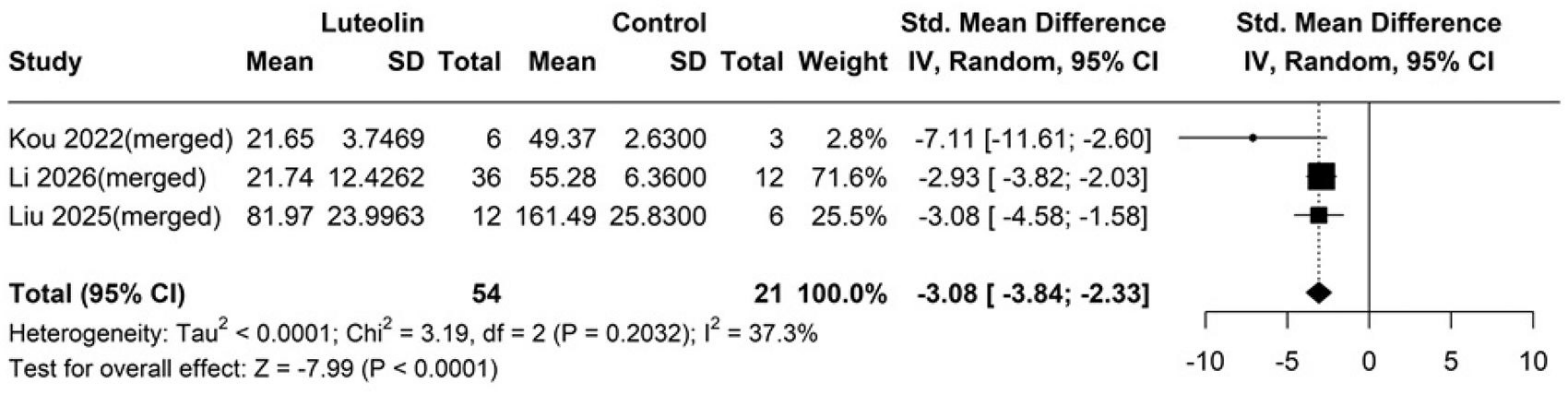
Meta-analysis of luteolin on IL-6 protein levels.

**Figure 14:**
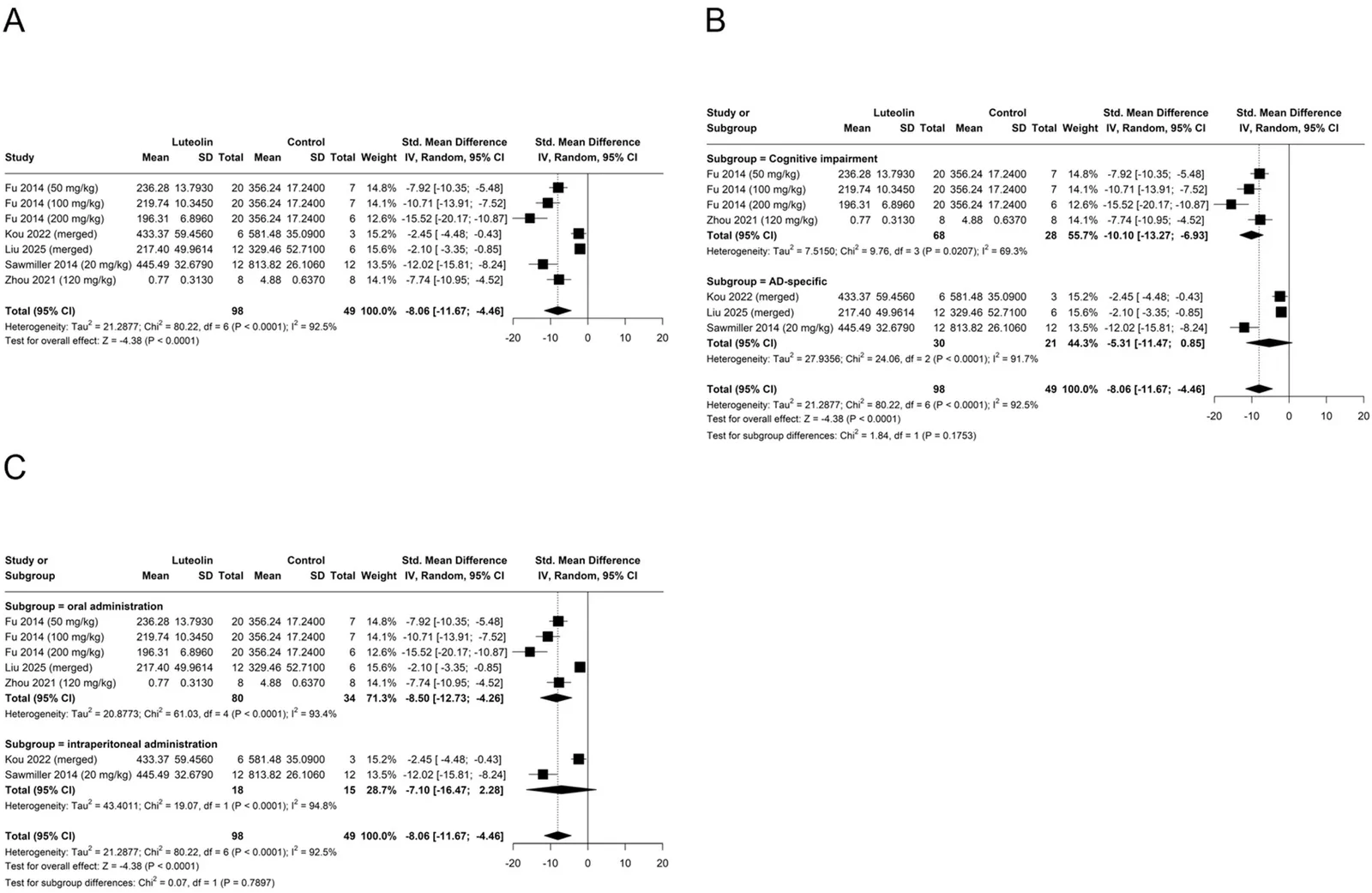
Meta-analysis and subgroup analyses of luteolin on TNF-α protein levels.

To rigorously investigate the sources of high heterogeneity in the ELISA data, multidimensional predefined subgroup analyses were conducted. First, to evaluate the impact of etiological diversity, datasets reporting IL-1β and TNF-α protein levels were stratified into AD-specific models and cognitive impairment models (this stratification was omitted for IL-6 due to insufficient dataset availability). For IL-1β, luteolin significantly reduced protein secretion in both AD-specific models (SMD = −2.76, 95% CI: [−4.23, −1.28]) and cognitive impairment models (SMD = −15.38, 95% CI: [−22.76,−7.99]) (subgroup difference *P* = 0.0010). For TNF-α, luteolin significantly reduced protein levels in cognitive impairment models (SMD = −10.10, 95% CI: [−13.27, −6.93]), whereas the pooled effect in AD-specific models showed a reductive trend but did not reach statistical significance (SMD = −5.31, 95% CI: [−11.47, 0.85]; subgroup difference *P* = 0.1753). Crucially, when conducting the subsequent subgroup analysis based on the “route of administration” we observed profound study-level confounding that overlaps extensively with the disease model classifications. Specifically, for IL-1β protein levels, the studies utilizing oral administration were perfectly identical to those in the cognitive impairment subgroup, while the intraperitoneal studies were identical to the AD-specific subgroup. This resulted in the exact same pooled effect sizes and subgroup difference (*P* = 0.0010) for both analytical dimensions. Similarly, for TNF-α protein levels, the oral administration subgroup maintained a significant inhibitory effect (SMD = −8.50, 95% CI: [−12.73, −4.26]), whereas the intraperitoneal subgroup crossed the line of no effect, closely mirroring the statistical pattern observed in the AD-specific subgroup (subgroup difference *P* = 0.7897). These overlapping findings definitively demonstrate that the magnitude of inflammatory cytokine suppression is heavily confounded by interrelated experimental variables. Consequently, while luteolin consistently suppresses neuroinflammation at the translational level, attempting to isolate the independent superiority of a specific administration route or a specific disease model is methodologically limited by these highly interconnected experimental designs.

Furthermore, during the methodological design phase, in-depth subgroup analyses regarding mRNA expression levels and “intervention duration” were initially planned. However, data verification revealed that all included mRNA studies utilized the oral route, and the stratification by intervention duration exhibited a high degree of single-source literature clustering (a single study encompassing multiple dose groups). To prevent false-positive conclusions arising from single-source confounding bias and insufficient statistical power, this study adhered strictly to the rigor of systematic reviews and did not subject the mRNA data or the intervention duration of cytokines to redundant quantitative subgroup partitioning. Taken together from this dual-dimensional analysis, luteolin can effectively modulate the neuroinflammatory network via a dual transcriptional and translational cascade.

#### 3.4.10 Assessment of publication bias and funnel plot analysis

Publication bias was systematically evaluated using visual inspection of funnel plots combined with Egger's regression test. Visual inspection revealed that the funnel plots for latency, target quadrant time, SOD, MDA, and inflammatory cytokines (both ELISA and mRNA) exhibited a certain degree of asymmetry, with several studies demonstrating significant dispersion outside the funnel regions. For primary outcomes with ≥10 datasets (i.e., latency, target quadrant time, and SOD), Egger's regression test quantitatively confirmed statistically significant funnel plot asymmetry (*P* < 0.05), indicating the presence of potential publication bias (Table 2, Supplementary Materials 2).

**Table 2:** Egger's test for publication bias.

Outcome	*t*	*p*	95% CI	No. of studies (containing different doses)
Morris water maze escape latency	−7.38	0.000	[−10.12, −5.47]	13
Morris water maze target quadrant time	9.77	0.000	[5.97, 9.65]	10
SOD levels	4.3	0.003	[2.84, 9.42]	10

It is important to comprehensively acknowledge that funnel plot asymmetry can arise from multiple interconnected factors. While significant methodological heterogeneity undoubtedly contributes to the broad distribution of effect sizes, this asymmetry cannot be solely dismissed as methodological variance. Other critical factors, notably small-study effects (where smaller studies demonstrating larger, positive effect sizes are preferentially published) and true publication bias (the selective non-reporting of negative findings), are highly likely to be present in this field. Consequently, there is a substantial risk that the overall pooled effect sizes calculated in this meta-analysis may be overestimated. The therapeutic magnitude of luteolin must therefore be interpreted with appropriate caution, taking this inherent statistical uncertainty into account.

#### 3.4.11 Sensitivity analysis

To examine the robustness of the meta-analysis findings and evaluate the potential influence of individual studies on the overall pooled effect size, a leave-one-out sensitivity analysis was performed by sequentially omitting each included study. The results demonstrated that the overall pooled effect sizes and statistical significance for all primary and secondary outcomes, including Morris water maze escape latency, target quadrant time, platform crossings, novel object recognition index, passive avoidance step-through latency, oxidative stress markers (SOD, MDA, GSH), and neuroinflammatory cytokines (IL-1β, IL-6, TNF-α at both protein and mRNA levels), did not experience directional reversal or qualitative alteration upon the exclusion of any single study. This indicates that the comprehensive conclusions of this study are stable, and are not disproportionately driven by any single independent study (Supplementary Materials 3).

## 4 Discussion

### 4.1 Summary of evidence

This systematic review and meta-analysis of 15 preclinical studies provide comprehensive evidence that luteolin exerts significant neuroprotective effects against cognitive impairment. Crucially, our subgroup analyses confirmed that these therapeutic benefits are consistent across both AD-specific amyloidogenic models and broader non-AD models of cognitive decline, effectively ameliorating deficits in spatial navigation, non-spatial recognition, and associative memory. Mechanistically, this broad-spectrum efficacy is driven by the significant restoration of redox homeostasis (characterized by the upregulation of SOD and GSH, and the reduction of MDA) alongside a dual-dimensional suppression of neuroinflammatory cascades (IL-1β, IL-6, and TNF-α) at both the transcriptional and translational levels.

Furthermore, our stratified analyses revealed important study-level characteristics: oral administration of luteolin is associated with significant *in vivo* efficacy, and larger effect sizes were generally observed in studies utilizing longer intervention durations (exceeding four weeks). However, as transparently identified in our cytokine subgroup analyses, these parameters are heavily confounded by interrelated experimental designs (such as the overlapping of specific disease models with specific administration routes). Consequently, while the therapeutic directionality is clear, these observational differences do not conclusively establish the pharmacokinetic superiority of the oral route or a time-dependent cumulative pharmacological effect. Supported by extensive sensitivity analyses that confirm the stability of these core findings, the current preclinical evidence underscores the translational potential of luteolin as a pleiotropic, symptom-alleviating, and neuroprotective candidate for AD and related cognitive impairments.

### 4.2 Potential mechanisms of luteolin in treating AD

The principal findings of this meta-analysis quantitatively establish that luteolin exerts highly consistent neuroprotective effects against AD-related cognitive impairment, primarily driven by the robust restoration of oxidative stress homeostasis and the suppression of selected neuroinflammatory cytokine cascades. However, to comprehensively map luteolin's broader neuropharmacological profile, it is imperative to clearly distinguish the mechanisms quantitatively validated in our meta-analysis from those reported by individual experimental studies.

Beyond the core meta-analyzed targets, a narrative synthesis of the included preclinical evidence suggests that luteolin may also act as a pleiotropic modulator involved in A*β* clearance, abnormal tau phosphorylation, PPAR*γ* and Nrf2 activation, autophagy, PI3K/Akt and insulin signaling, as well as cholinergic and synaptic functions. It must be strongly emphasized that these additional pathways were not quantitatively meta-analyzed in the current study due to the limited number of comparable datasets. Consequently, while supported by individual studies, these pathways should be strictly interpreted as promising mechanistic hypotheses rather than meta-analytically established conclusions. Despite the significant etiological heterogeneity of the included animal models—encompassing A*β* burden, chronic cerebral hypoperfusion, inflammatory stimulation, aging, and traumatic brain injury—this cross-model consistency further supports the potential translational value of luteolin as a multi-mechanism neuroprotective candidate (Figure 15).

**Figure 15:**
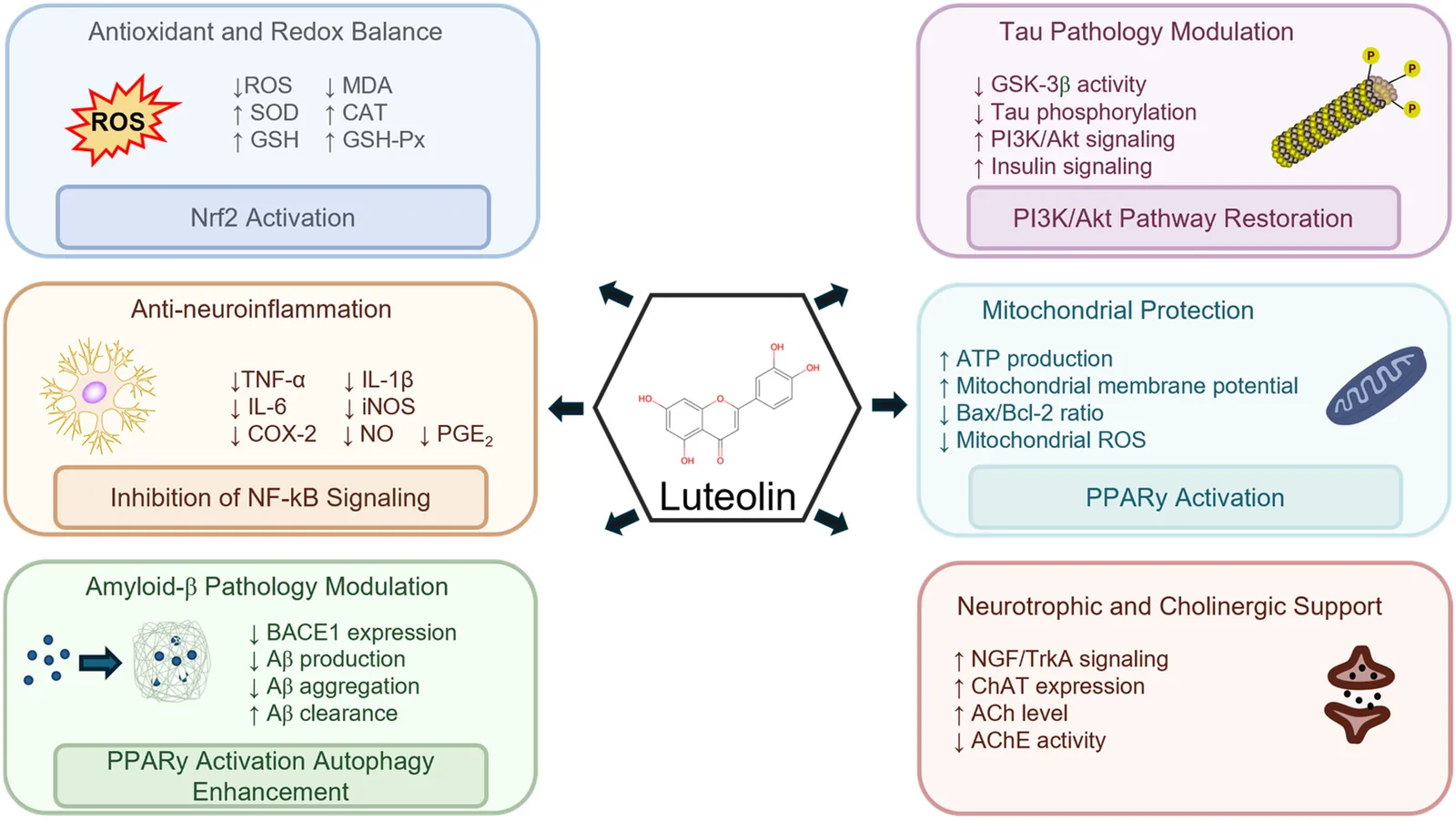
Schematic representation of the neuroprotective mechanisms of luteolin against Alzheimer's disease.

The inhibition of oxidative stress and the maintenance of mitochondrial homeostasis may constitute the foundation of luteolin's neuroprotective effects. Oxidative stress is a critical network hub connecting A*β* toxicity, neuroinflammation, mitochondrial dysfunction, and neuronal death (30, 31). In chronic cerebral hypoperfusion models, luteolin significantly reduced lipid peroxidation in the cortex and hippocampus while restoring antioxidant capacity; similar effects were observed in traumatic brain injury (TBI) and A*β*-induced neuronal injury models, suggesting that the restoration of redox homeostasis is a relatively stable common mechanism (16, 18). This antioxidant effect does not solely stem from its chemical properties as a direct free radical scavenger. Mechanistic studies indicate that luteolin promotes the nuclear translocation of Nrf2 and upregulates the expression of antioxidant defense proteins such as HO-1 and NQO1; conversely, its neuroprotective effects are significantly diminished in Nrf2-deficient animals, indicating that the Nrf2-mediated endogenous antioxidant response is profoundly involved in its mechanistic network (32, 33). Furthermore, in the 3 × Tg-AD mouse model, luteolin improved A*β*-associated oxidative stress, stabilized mitochondrial membrane potential, and reduced cellular apoptosis, whereas pharmacological blockade or genetic silencing of PPAR*γ* essentially abolished these protective effects. This provides crucial evidence of a specific molecular dependency for its antioxidant and mitochondria-protecting properties (18).

Regarding the alleviation of chronic neuroinflammation, the modulation of microglial and astrocytic activation represents a core mechanism (34). In *in vitro* LPS-stimulated microglia and *in vivo* aged mouse models, luteolin inhibited the expression of IL-1β and TNF-α in a concentration-dependent manner and restored hippocampal-dependent spatial working memory, supporting its role in conferring cognitive protection through the modulation of glial activation (19). At the signal transduction level, NF-*κ*B appears to be a crucial hub within this inflammatory network. Research using the chronic cerebral hypoperfusion model confirmed that by inhibiting NF-*κ*B activation, luteolin not only reduced the expression of TNF-α and IL-1β but also concomitantly decreased BACE1 expression and A*β* burden (16). Additionally, endoplasmic reticulum stress may lie between aberrant glial activation and inflammatory signaling. In 3 × Tg-AD mice, luteolin significantly reduced GRP78 and IRE1*α* expression, inhibited astrocytic overactivation, and blocked NF-*κ*B and p38 inflammatory signaling pathways (20). This indicates that luteolin can multidimensionally modulate the pathological state of glial cells, thereby severing the toxic damage inflicted on neurons by persistent inflammatory signaling.

Against A*β* amyloid pathology, luteolin exhibits a dual regulatory effect: reducing production and promoting cellular clearance. In rats with chronic cerebral hypoperfusion, luteolin inhibited NF-*κ*B activation, which subsequently reduced BACE1 expression and attenuated the accumulation of A*β*_1–40_ and A*β*_1–42_ in brain tissue (16). In the Tg2576 mouse TBI model, luteolin not only significantly curbed the surge of soluble A*β*_40_ and A*β*_42_, but also synchronously mitigated GSK-3 activation and abnormal tau phosphorylation (25). A recent 3 × Tg-AD study further revealed that luteolin can concurrently decrease A*β* generation and promote its degradation, an effect that is also closely linked to PPAR*γ* activity (18). Furthermore, cellular autophagy may constitute another vital link in its maintenance of protein quality control. In a study combining luteolin intervention with exercise, autophagy-related markers (Beclin1 and LC3-II) were restored and abnormal p62 accumulation was reduced in AD mice, whereas autophagy inhibitors partially negated the improvements in cognition and neurogenesis brought about by this combined intervention (26).

Aside from A*β*, the hyperphosphorylation of tau protein is another key feature driving neurodegeneration in AD (35). Current evidence suggests that luteolin's regulation of tau pathology may be intimately connected to the PI3K/Akt, GSK-3β, and insulin signaling cascades. In models of chronic ischemia and TBI, luteolin restored PI3K/Akt activity, reduced abnormally activated GSK-3β, and significantly downregulated tau phosphorylation levels, thereby improving cognitive performance (17, 25). This finding aligns closely with the pathophysiological characteristics of brain insulin resistance in AD (36). In a rat model induced by A*β* combined with a high-fat diet, luteolin markedly enhanced the pAkt, pGSK-3β, pFOXO1, and pCREB insulin signaling cascade in the hippocampus; it decreased tau hyperphosphorylation while simultaneously improving systemic and hepatic insulin sensitivity (24). Taken together, the PI3K/Akt pathway appears to be an essential intersection linking metabolic homeostasis, tau phosphorylation, neuronal survival, and cognitive function, positioning it as a prominent candidate mediating pathway for luteolin's amelioration of AD-like pathology.

Beyond alleviating pathological burden, luteolin may also directly support cognitive recovery by improving neurotrophic support, cholinergic transmission, and synaptic plasticity. In an A*β*-induced AD model, luteolin not only elevated acetylcholine (ACh) levels and modulated AChE and ChAT activities, but also ameliorated the apoptotic propensity caused by Bcl-2/Bax imbalance (27). In a scopolamine-induced amnesia model, luteolin similarly demonstrated the potential to improve spatial memory, reduce lipid peroxidation, and promote hippocampal neurogenesis (37). These findings imply that the cognitive benefits of luteolin derive not solely from the clearance of pathological markers, but likely also depend on an overall enhancement of synaptic plasticity and the reparative capacity of damaged neural networks.

Integrating these multidimensional mechanisms, the complex progression of AD triggers oxidative stress and disrupts organelle homeostasis, which in turn amplifies aberrant glial activation, NF-*κ*B inflammatory responses, and tau phosphorylation. Luteolin appears capable of acting concurrently on multiple mutually amplifying pathological nodes within this network. On one hand, it lowers the burden of oxidative stress and chronic inflammation via key regulatory nodes such as PPAR*γ*, Nrf2, and NF-*κ*B; on the other hand, it maintains neuronal survival and synaptic function through the PI3K/Akt pathway, brain insulin signaling, the autophagy network, and cholinergic mechanisms. Related pharmacological reviews have similarly emphasized the neuroprotective profile of luteolin involving multiple interacting pathways, including Nrf2, NF-*κ*B, and MAPK (38, 39). This dual regulation, encompassing both anti-burden and pro-homeostasis properties, showcases its pleiotropic advantages.

### 4.3 Pharmacokinetic barriers and safety considerations

While comprehensively elucidating the multi-target mechanisms of luteolin against AD pathology, it is imperative to critically evaluate its clinical translational potential from a macroscopic pharmacokinetic (PK) and toxicological perspective. For any prospective therapeutic agent targeting neurodegenerative diseases, overcoming the BBB is a fundamental prerequisite (40). As a lipophilic small-molecule flavonoid, luteolin has been experimentally demonstrated to cross the BBB. Specifically, recent rigorous *in vitro* quantitative assessments utilizing co-cultured BBB models have confirmed its significant permeability, yielding an apparent permeability coefficient (*P*_app_) of approximately 10 × 10^−6^ cm/s. While this establishes the essential physical foundation for its distribution into critical brain regions, it is crucial to clarify that achieving absolute “therapeutically active” concentrations *in vivo* via conventional systemic administration has not been definitively established. Because continuous *in situ* pharmacokinetic monitoring is largely absent in current preclinical efficacy trials, whether the absolute brain exposure levels independently drive the observed phenotypic improvements remains a critical gap requiring future quantitative PK validation (12, 41).

However, the findings of our meta-analysis present a compelling paradox. Conventional pharmacological consensus maintains that luteolin exhibits exceedingly low absolute oral bioavailability due to its poor aqueous solubility and extensive first-pass metabolism (42). Our subgroup analyses revealed that studies utilizing oral administration reported significant *in vivo* effect sizes in ameliorating core pathological hallmarks. However, it is crucial to emphasize that a larger pooled SMD in orally treated animals does not inherently demonstrate better bioavailability or pharmacokinetic superiority. In our meta-analysis, the administration route is observationally confounded by other study-level characteristics, including discrepancies in drug doses, animal models, intervention durations, and specific formulations. Therefore, pharmacokinetic properties cannot be directly inferred from these behavioral or biochemical effect sizes (43). To theoretically reconcile this discrepancy, we propose a pharmacokinetic hypothesis based on broader inflammation models, though its specific application to AD remains highly speculative. On one hand, while the abundant circulating plasma metabolites are relatively inactive under physiological conditions, it is hypothesized that the severe neuroinflammatory microenvironment in AD brain lesions might alter local metabolite dynamics. Hyperactivated microglia and astrocytes in inflammatory lesions have been reported in other contexts to express *β*-glucuronidase, an enzyme capable of hydrolyzing these inactive conjugates back into the free, active luteolin aglycone. This mechanism could theoretically facilitate an inflammation-driven, “targeted local drug release” precisely at the site of neurodegeneration (44). However, direct PK/PD validation tracking luteolin metabolite conversion *in situ* within AD brain lesions is strictly required before this concept can be accepted as a definitive explanation for our findings. On the other hand, the unabsorbed luteolin remaining in the gastrointestinal tract might not be functionally void; it has been proposed to act as a targeted prebiotic to reshape gut microbiota dysbiosis, potentially alleviating the systemic inflammatory burden via the “microbiota-gut-brain axis” thereby synergistically exerting central neuroprotective effects (13, 45).

While establishing its pharmacokinetic translational potential is crucial, advancing luteolin toward clinical application mandates a highly rigorous and systematic toxicological evaluation (46). Previously, it has often been noted that luteolin's ubiquitous distribution in dietary vegetables and its high acute median lethal dose (LD50 > 5,000 mg/kg in rodents) suggest a broad safety margin (47). However, it is imperative to clarify that routine dietary exposure is fundamentally inequivalent to chronic, targeted pharmacological dosing. Furthermore, high acute LD50 values only reflect acute tolerability and do not rule out the potential for cumulative, organ-specific subclinical toxicities during prolonged administration. Similarly, although no significant adverse events were explicitly reported among the rodent studies included in our meta-analysis, this absence of reporting cannot be scientifically interpreted as evidence of safety, given the small sample sizes, brief observation windows, and lack of systematic toxicological screening inherent to these efficacy-focused preclinical models. Consequently, rigorous, Good Laboratory Practice-compliant longitudinal toxicological profiling—specifically evaluating chronic exposure limits, potential off-target effects, and systemic tolerability—is strictly required before definitively claiming safety or advancing to human clinical trials.

Despite these encouraging macroscopic safety profiles, adhering to rigorous pharmacological standards requires acknowledging potential subclinical toxicities and condition-specific adverse reactions. *In vitro* mechanistic studies suggest that flavonoids at ultra-high dose exposures may undergo an “antioxidant-prooxidant paradox”, paradoxically inducing the collapse of mitochondrial membrane potential and exacerbating oxidative stress (48).

### 4.4 Limitations and future perspectives

While this systematic review reveals the compelling neuroprotective potential of luteolin, it is essential to objectively acknowledge some inherent limitations in the current preclinical evidence base. First, the methodological quality of the included studies is significantly deficient. Most preclinical trials lack detailed reporting on random sequence generation, allocation concealment, and blinding outcome assessment, introducing potential selection and detection biases. Furthermore, the lack of standardized, universally adopted semi-quantitative criteria for assessing core pathological features also contributes to methodological heterogeneity. Second, the animal models used suffer from severe translational limitations. Although the included studies cover multiple etiologies, no transgenic or inducible rodent model fully reproduces the insidious, protracted, and multifactorial pathological evolution of sporadic human AD. Third, quantitative assessments revealed significant funnel plot asymmetry for several primary outcomes. This asymmetry highlights the probable existence of publication bias and small-study effects within the current literature, suggesting a systemic tendency to selectively publish positive experimental findings. Consequently, evaluating the overall certainty of the current preclinical evidence reveals inherent limitations. Although a formal GRADE-adapted grading table was not constructed, the widespread “unclear” risk of bias in key methodological domains, coupled with substantial statistical heterogeneity (*I*^2^ frequently >80%) and potential publication bias, inherently downgrades the confidence level of the pooled effect sizes. These factors indicate that while the therapeutic trends are consistent, the absolute magnitude of luteolin's efficacy is likely prone to overestimation, necessitating a highly cautious interpretation.

Mechanistically, while pharmacological blockade and gene manipulation studies provide strong causal evidence, research on other emerging pathways remains scarce. Current mechanistic evidence primarily focuses on phenotypic relevance rather than explicit direct target binding. The precise molecular binding target of luteolin, and the distinction between primary receptor interactions and secondary cascade network effects, remain incompletely elucidated. Furthermore, the extremely large magnitude of several pooled effect sizes requires cautious interpretation. Such extreme estimates, while mathematically accurate based on the extracted data, do not directly translate to proportional clinical efficacy. In preclinical meta-analyses, these inflated SMDs are frequently mathematical artifacts driven by the genetic homogeneity of inbred animal strains and highly controlled laboratory environments. These factors routinely yield exceptionally small standard deviations in experimental control groups, drastically inflating the SMD denominator. Therefore, while our leave-one-out sensitivity analyses confirm that the direction of luteolin's neuroprotective effect is highly robust, the absolute magnitude of these extreme SMDs reflects preclinical experimental characteristics and should not be directly extrapolated to human clinical expectations.

From a translational and toxicological perspective, several key hurdles remain unresolved. Existing evidence is almost entirely derived from short- to medium-term interventions in rodent models. Although luteolin has a good baseline safety profile and has been shown to cross the BBB, its absolute systemic exposure and potential accumulation in vital organs after long-term chronic administration require rigorous longitudinal assessment. Moreover, there is a significant lack of preclinical data on potential synergistic effects or drug interactions between luteolin and standard antidementia medications.

Looking ahead, to bridge the translational gap from laboratory to clinic, future preclinical studies must strictly adhere to the SYRCLE and ARRIVE guidelines to ensure rigorous experimental design, minimize bias, and guarantee transparent reporting. Mechanistically, there is an urgent need to integrate cutting-edge multi-omics technologies—such as single-cell transcriptomics, spatial proteomics, and conditional gene knockout—to precisely resolve the multi-target interaction genome of luteolin. From a pharmacokinetic perspective, future research should prioritize the development of advanced brain-targeted drug delivery systems to overcome systemic exposure bottlenecks. Once these advanced formulations can maximize bioavailability, systematic chronic toxicology studies using large animal models must be conducted under Good Laboratory Practice conditions. Ultimately, accurately defining the dynamic safety profile and drug interaction risks of luteolin in elderly patients with multiple comorbidities will be a primary prerequisite for initiating well-designed early clinical trials, thereby fully evaluating the potential of luteolin as a symptom-alleviating and neuroprotective therapy for AD.

## 5 Conclusion

In conclusion, this systematic review and meta-analysis provides encouraging preclinical evidence suggesting that luteolin has the potential to ameliorate cognitive and memory impairments in AD models. These benefits appear to be driven by its multi-target ability to suppress neuroinflammation, restore oxidative balance, and mitigate A*β* and tau pathologies. Crucially, our findings indicate that oral administration of luteolin exhibits promising *in vivo* efficacy, with neuroprotective benefits potentially increasing over prolonged treatment durations. However, due to prominent methodological limitations across the included studies—most notably the substantial heterogeneity and the probable existence of publication bias and small-study effects that may inflate pooled effect sizes—the current conclusions must be interpreted with caution. While luteolin represents a promising neuroprotective candidate for AD, its true translational value and absolute therapeutic magnitude rely on future rigorous, large-scale, and transparently reported preclinical evaluations and toxicological assessments before progressing to human clinical trials.
