# Supplementary material for: Luteolin for Alzheimer's disease and related cognitive impairment: a systematic review and meta-analysis of efficacy and molecular mechanisms
Source: Front Med (Lausanne). 2026 Oct 14;13:1976119. doi: 10.3389/fmed.2026.1976119 (PMC13714536; doi:10.3389/fmed.2026.1976119)
Supplement: Supplementary file 1 [file Table1.docx]

Supplementary Materials 1

Search strategy in 8 Databases ((PubMed, Embase, Web of Science, Cochrane Library, CNKI, Wanfang, VIP, and Chinese Biomedical Database), and they are presented respectively in the form of tables.

1 Search strategy in PubMed

| **Search ID** | **Concept** | **Search Query** |
| --- | --- | --- |
| **#1** | **Intervention** | "Luteolin"[Mesh] OR "luteolin"[Title/Abstract] OR "luteolol"[Title/Abstract] OR "3',4',5,7-Tetrahydroxyflavone"[Title/Abstract] |
| **#2** | **Disease/Condition** | "Alzheimer Disease"[Mesh] OR "Brain Diseases"[Mesh] OR "Cognitive Dysfunction"[Mesh] OR "Memory Disorders"[Mesh] OR "Alzheimer*"[Title/Abstract] OR "dementia"[Title/Abstract] OR "encephalopath*"[Title/Abstract] OR "cognitive impairment"[Title/Abstract] OR "cognitive dysfunction"[Title/Abstract] OR "cognitive decline"[Title/Abstract] OR "memory impairment"[Title/Abstract] OR "memory loss"[Title/Abstract] |
| **#3** | **Study Design** | "Disease Models, Animal"[Mesh] OR "Animals"[Mesh] OR "In Vitro Techniques"[Mesh] OR "Cells, Cultured"[Mesh] OR "animal*"[Title/Abstract] OR "mice"[Title/Abstract] OR "mouse"[Title/Abstract] OR "rat"[Title/Abstract] OR "rats"[Title/Abstract] OR "murine"[Title/Abstract] OR "rodent*"[Title/Abstract] OR "in vitro"[Title/Abstract] OR "in vivo"[Title/Abstract] OR "cell"[Title/Abstract] OR "cells"[Title/Abstract] OR "preclinical"[Title/Abstract] OR "mechanism*"[Title/Abstract] |
| **#4** | **Exclusions** | "Clinical Trials as Topic"[Mesh] OR "Clinical Trial"[Publication Type] OR ("humans"[MeSH Terms] NOT "animals"[MeSH Terms]) |
| **#5** | **Final Combination** | **#1 AND #2 AND #3 NOT #4** |

2 Search strategy in Embase

| **Search ID** | **Concept** | **Search Query** |
| --- | --- | --- |
| **#1** | **Intervention** | ('luteolin'/exp OR luteolin:ti,ab OR luteolol:ti,ab OR '3,4,5,7 tetrahydroxyflavone':ti,ab) |
| **#2** | **Disease/Condition** | ('Alzheimer disease'/exp OR 'dementia'/exp OR 'brain disease'/exp OR 'cognitive defect'/exp OR 'memory disorder'/exp OR Alzheimer*:ti,ab OR dementia:ti,ab OR encephalopath*:ti,ab OR 'cognitive impairment':ti,ab OR 'cognitive dysfunction':ti,ab OR 'cognitive decline':ti,ab OR 'memory impairment':ti,ab OR 'memory loss':ti,ab) |
| **#3** | **Study Design** | ('animal model'/exp OR 'animal'/exp OR 'in vitro study'/exp OR 'cell culture'/exp OR animal*:ti,ab OR mice:ti,ab OR mouse:ti,ab OR rat:ti,ab OR rats:ti,ab OR murine:ti,ab OR rodent*:ti,ab OR 'in vitro':ti,ab OR 'in vivo':ti,ab OR preclinical:ti,ab OR cell*:ti,ab OR mechanism*:ti,ab) |
| **#4** | **Exclusions** | ('clinical trial'/exp OR 'human'/exp NOT 'animal'/exp) |
| **#5** | **Final Combination** | **#1 AND #2 AND #3 NOT #4** |

3 Search strategy in WOS

| **Search ID** | **Concept** | **Search Query** |
| --- | --- | --- |
| **#1** | **Intervention** | TS=("luteolin" OR "luteolol" OR "3',4',5,7-Tetrahydroxyflavone") |
| **#2** | **Disease/Condition** | TS=("Alzheimer*" OR "dementia" OR "encephalopath*" OR "cognitive impairment" OR "cognitive dysfunction" OR "cognitive decline" OR "memory impairment" OR "memory loss") |
| **#3** | **Study Design** | TS=("animal*" OR "mice" OR "mouse" OR "rat" OR "rats" OR "murine" OR "rodent*" OR "in vitro" OR "in vivo" OR "preclinical" OR "cell*" OR "mechanism*") |
| **#4** | **Exclusions** | TS=("clinical trial*" OR "randomized controlled trial" OR "case report*" OR "patients") |
| **#5** | **Final Combination** | **#1 AND #2 AND #3 NOT #4** |

4 Search strategy in Cochrane Library

| **Search ID** | **Concept** | **Search Query** |
| --- | --- | --- |
| **#1** | **Intervention** | ("luteolin" OR "luteolol" OR "3',4',5,7-Tetrahydroxyflavone"):ti,ab,kw |
| **#2** | **Disease/Condition** | ("Alzheimer*" OR "dementia" OR "encephalopath*" OR "cognitive impairment" OR "cognitive dysfunction" OR "cognitive decline" OR "memory impairment" OR "memory loss"):ti,ab,kw |
| **#3** | **Study Design** | ("animal*" OR "mice" OR "mouse" OR "rat" OR "rats" OR "murine" OR "rodent*" OR "in vitro" OR "in vivo" OR "preclinical" OR "cell*" OR "mechanism*"):ti,ab,kw |
| **#4** | **Final Combination** | **#1 AND #2 AND #3** |

5 Search strategy in CNKI

| **Search ID** | **Concept** | **Search Query** |
| --- | --- | --- |
| **#1** | **Intervention** | (SU%=木犀草素 OR SU%=黄酮类木犀草素 OR SU%=3',4',5,7-四羟基黄酮 OR TKA=木犀草素) |
| **#2** | **Disease/Condition** | (SU%=阿尔茨海默病 OR SU%=阿尔茨海默症 OR SU%=老年痴呆 OR SU%=痴呆 OR SU%=认知障碍 OR SU%=认知功能障碍 OR SU%=记忆障碍 OR SU%=脑病) |
| **#3** | **Study Design** | (SU%=动物模型 OR SU%=小鼠 OR SU%=大鼠 OR SU%=啮齿类 OR SU%=体外实验 OR SU%=体内实验 OR SU%=细胞 OR SU%=机制 OR SU%=实验研究 OR SU%=基础研究) |
| **#4** | **Exclusions** | (SU%=临床试验 OR SU%=临床观察 OR SU%=病例报告 OR SU%=临床疗效 OR SU%=患者) |
| **#5** | **Final Combination** | **#1 AND #2 AND #3 NOT #4** |

6 Search strategy in Wanfang Database

| **Search ID** | **Concept** | **Search Query** |
| --- | --- | --- |
| **#1** | **Intervention** | (主题:(木犀草素) OR 主题:(3',4',5,7-四羟基黄酮) OR 题名或摘要:(木犀草素)) |
| **#2** | **Disease/Condition** | (主题:(阿尔茨海默病) OR 主题:(老年痴呆) OR 主题:(痴呆) OR 主题:(认知障碍) OR 主题:(认知功能障碍) OR 主题:(记忆障碍) OR 主题:(脑病)) |
| **#3** | **Study Design** | (主题:(动物模型) OR 主题:(小鼠) OR 主题:(大鼠) OR 主题:(啮齿) OR 主题:(体外) OR 主题:(体内) OR 主题:(细胞) OR 主题:(机制) OR 主题:(实验) OR 主题:(基础研究)) |
| **#4** | **Exclusions** | (主题:(临床试验) OR 主题:(临床观察) OR 主题:(病例报告) OR 主题:(疗效观察) OR 主题:(患者)) |
| **#5** | **Final Combination** | **#1 AND #2 AND #3 NOT #4** |

7 Search strategy in VIP Database

| **Search ID** | **Concept** | **Search Query** |
| --- | --- | --- |
| **#1** | **Intervention** | (M=木犀草素 OR M=3',4',5,7-四羟基黄酮) |
| **#2** | **Disease/Condition** | (M=阿尔茨海默病 OR M=阿尔茨海默症 OR M=老年痴呆 OR M=痴呆 OR M=认知障碍 OR M=认知功能障碍 OR M=记忆障碍 OR M=脑病) |
| **#3** | **Study Design** | (M=动物模型 OR M=小鼠 OR M=大鼠 OR M=啮齿 OR M=体外 OR M=体内 OR M=细胞 OR M=机制 OR M=实验研究 OR M=基础研究) |
| **#4** | **Exclusions** | (M=临床试验 OR M=临床观察 OR M=病例报告 OR M=疗效观察 OR M=患者) |
| **#5** | **Final Combination** | **#1 AND #2 AND #3 NOT #4** |

8 Search strategy in CBM

| **Search ID** | **Concept** | **Search Query** |
| --- | --- | --- |
| **#1** | **Intervention** | "木犀草素"[不加权:扩展] OR "木犀草素"[常用字段:智能] OR "3',4',5,7-四羟基黄酮"[常用字段:智能] |
| **#2** | **Disease/Condition** | "阿尔茨海默病"[不加权:扩展] OR "痴呆"[不加权:扩展] OR "脑疾病"[不加权:扩展] OR "老年痴呆"[常用字段:智能] OR "认知障碍"[常用字段:智能] OR "记忆障碍"[常用字段:智能] OR "脑病"[常用字段:智能] |
| **#3** | **Study Design** | "疾病模型, 动物"[不加权:扩展] OR "动物"[不加权:扩展] OR "细胞, 培养的"[不加权:扩展] OR "小鼠"[常用字段:智能] OR "大鼠"[常用字段:智能] OR "啮齿类"[常用字段:智能] OR "体外研究"[常用字段:智能] OR "体内研究"[常用字段:智能] OR "细胞"[常用字段:智能] OR "机制"[常用字段:智能] |
| **#4** | **Exclusions** | 临床试验[文献类型] OR 病例报告[文献类型] OR 临床观察[常用字段:智能] OR 患者[常用字段:智能] OR (人类[特征词] NOT 动物[特征词]) |
| **#5** | **Final Combination** | **(#1) AND (#2) AND (#3) NOT (#4)** |
