# Supplementary material for: Luteolin for Alzheimer's disease and related cognitive impairment: a systematic review and meta-analysis of efficacy and molecular mechanisms
Source: Front Med (Lausanne). 2026 Oct 14;13:1976119. doi: 10.3389/fmed.2026.1976119 (PMC13714536; doi:10.3389/fmed.2026.1976119)
Supplement: Supplementary file 2 [file Table2.docx]

Supplementary Materials 2

Supplementary Materials 2. Funnel plots for the visual inspection of potential publication bias.

Each point in the funnel plots represents an independent dataset from the included studies. The plots display the distribution of effect sizes for the following outcome measures: (A) Morris water maze escape latency; (B) Morris water maze target quadrant time; (C) Morris water maze platform crossings; (D) Passive avoidance (PA) step-through latency; (E) Novel object recognition index; (F) SOD levels; (G) GSH levels; (H) MDA levels; (I) TNF-α protein levels; (J) TNF-α mRNA expression; (K) IL-1β protein levels; (L) IL-1β mRNA expression; and (M) IL-6 protein levels.
