# Supplementary material for: Luteolin for Alzheimer's disease and related cognitive impairment: a systematic review and meta-analysis of efficacy and molecular mechanisms
Source: Front Med (Lausanne). 2026 Oct 14;13:1976119. doi: 10.3389/fmed.2026.1976119 (PMC13714536; doi:10.3389/fmed.2026.1976119)
Supplement: Supplementary file 3 [file Table3.docx]

Supplementary Materials 3

Supplementary Materials 3. Leave-one-out sensitivity analysis for evaluating the robustness of the meta-analysis results.

(A) Morris water maze escape latency

(B) Morris water maze target quadrant time

(C) Morris water maze platform crossings

(D) Passive avoidance (PA) step-through latency

(E) Novel object recognition index

(F) SOD levels

(G) GSH levels

(H) MDA levels

(I)TNF-α protein levels

(J) TNF-α mRNA expression

(K) IL-1β protein levels

(L) IL-1β mRNA expression

(M) IL-6 protein levels
